# Sarcopenic Obesity and Outcomes for Patients With Cancer

**DOI:** 10.1001/jamanetworkopen.2024.17115

**Published:** 2024-06-14

**Authors:** Chenan Liu, Tong Liu, Li Deng, Qi Zhang, Mengmeng Song, Jinyu Shi, Chenning Liu, Hailun Xie, Yue Chen, Shiqi Lin, Xin Zheng, Heyang Zhang, Rocco Barazzoni, Hanping Shi

**Affiliations:** 1 Department of Gastrointestinal Surgery/Clinical Nutrition, Beijing Shijitan Hospital, Capital Medical University, Beijing, China; 2 National Clinical Research Center for Geriatric Diseases, Xuanwu Hospital, Capital Medical University, Beijing, China; 3 Key Laboratory of Cancer FSMP for State Market Regulation, Beijing, China; 4 Laboratory for Clinical Medicine, Capital Medical University, Beijing, China; 5 Department of Genetics, Yale University School of Medicine, New Haven; 6 Cardiovascular Research Institute, University of California, San Francisco; 7 State Key Laboratory of Quality Research in Chinese Medicine, Institute of Chinese Medical Sciences, University of Macau, Macao, China; 8 Department of Medical, Surgical and Health Sciences, University of Trieste, Trieste, Italy

## Abstract

**Importance:**

The European Society for Clinical Nutrition and Metabolism (ESPEN) and the European Association for the Study of Obesity (EASO) have recently proposed a consensus definition and diagnostic criteria for sarcopenic obesity (SO).

**Objective:**

To implement the ESPEN-EASO diagnostic algorithm to investigate the prevalence of SO and its association with outcomes in patients with solid tumor cancers, with particular regard to associations among SO, overall survival (OS), and patient quality of life (QoL).

**Design, Setting, and Participants:**

This prospective cohort study included patients diagnosed with solid tumor starting in May 7, 2013, with the last follow-up on June 30, 2022. Patients with solid tumors were categorized into SO and non-SO groups according to ESPEN-EASO criteria. The primary outcome was OS and the secondary outcomes included patient QoL and risk of intensive care unit (ICU) admission. Data were analyzed from June to December 2023.

**Results:**

A total of 6790 patients were included in the study (mean [SD] age, 59.64 [10.77] years; 3489 were female [51.4%]). The prevalence of SO was 4.36% (296 of 6790) in the whole cohort and 14.98% (296 of 1976) in the subgroup with obesity. SO prevalence increased with age. During a median (IQR) follow-up period of 6.83 (5.67-7.04) years, 2103 patients died. Cox regression analysis indicated that SO was independently associated with lower OS (hazard ratio [HR], 1.54; 95% CI, 1.23-1.92), which was observed in both men (HR, 1.51; 95% CI, 1.09-2.10) and women (HR, 1.53; 95% CI, 1.12-2.07). SO was also associated with poorer QoL and higher risk of ICU admission (odds ratio, 2.39; 95% CI, 1.06-5.29). Among the diagnostic components of SO, low hand grip strength (HGS) was the only SO component associated with poor OS (HR, 1.15; 95% CI, 1.04-1.28).

**Conclusions and Relevance:**

This cohort study of SO found that SO was significantly associated with lower OS, poorer QoL, and higher risk of ICU admission. Weak HGS, 1 of the diagnostic conditions, was the only component of SO associated with OS. The ESPEN-EASO algorithm appears to be an applicable tool to identify cancer-associated SO, which represents a major clinical complication and factor associated with risk for poor outcomes in these patients.

## Key Points

**Question:**

Is sarcopenic obesity (SO) associated with outcomes in patients with solid tumor cancers?

**Findings:**

In this cohort study involving 6790 patients with cancer, the prevalence of SO was 4.36%. Cox regression analysis indicated that SO was associated with a greater than 50% higher risk of death in both male and female patients; among the diagnostic components of SO, low hand grip strength (HGS) was more profoundly associated with poor overall survival (OS).

**Meaning:**

These findings suggest SO was associated with poor OS of patients with cancer and should be integrated into clinical care of cancer patients.

## Teaser

This cohort study evaluates the prevalence of sarcopenic obesity and its association with outcomes in patients with solid tumor cancers.

## Introduction

Sarcopenic obesity (SO) is an emerging clinical condition characterized by the coexistence of obesity and low muscle mass and function.^1^ Sarcopenia was originally defined by the European Working Group on Sarcopenia in Older People 2 (EWGSOP2) in the geriatric population as a progressive systemic skeletal muscle disease that involves reduction of muscle mass and loss of muscle function, mainly represented by muscle strength.^2^ The prevalence of sarcopenia has, however, also more recently emerged before old age, particularly in the presence of chronic diseases.^3^ In patients with cancer, cancer-induced inflammation commonly leads to muscle catabolism and altered energy metabolism, making sarcopenia a common complication.^4^ After conducting thorough assessments of participants’ muscle mass and strength to define sarcopenia, some studies found that sarcopenia is associated with a poor quality of life (QoL) and poor outcomes in patients with cancer.^5,6^ Additionally, it is highly correlated with higher rates of severe chemotherapy toxic effects and related changes in body composition.^7^

Obesity, as another feature of SO, has also attracted much attention. Extensive research has established that obesity is closely related to an increased risk of cancer.^8^ Counterintuitively, in the context of patients with cancer, some studies have reported better cancer outcomes in the presence of high body mass index (BMI).^9^ However, when obesity and low muscle mass are combined, they have been consistently associated with negative impact on survival.^10^ In addition, although SO per se is not inevitably associated with frailty, obesity with low muscle mass may represent a risk factor for frailty and disability.^11^ It seems therefore particularly relevant to clarify the characteristics of SO and its potential association with outcomes in patients with cancer.

In 2022, the European Society for Clinical Nutrition and Metabolism (ESPEN) and the European Association for the Study of Obesity (EASO) initiated efforts to establish expert consensus on the definition and diagnostic criteria for SO.^12^ According to this consensus, SO should be rigorously screened, diagnosed, and staged. However, as for most new consensuses, the general applicability and cutoff values of parameters in complex clinical settings will require future validation and optimization. Therefore, this multicenter prospective cohort study aimed to evaluate the prevalence of SO diagnosed according to the ESPEN and EASO criteria and its association with survival and quality of life (QoL) in patients with cancer.

## Methods

### Study Design and Study Population

The study population was derived from the Investigation of Nutrition Status and its Clinical Outcome of Common Cancers (INSCOC), which was initiated by our research group in 2013.^13^ All patients provided their written informed consent at the time of enrolment. Inclusion and exclusion criteria for this study are described in the eMethods 1 in Supplement 1. Briefly, we included adult patients with solid tumors, such as lung, digestive system, female reproductive system, and breast cancer. This cohort study was approved by the ethics committee of Beijing Shijitan Hospital and was reported following the Strengthening the Reporting of Observational Studies in Epidemiology (STROBE) reporting guideline. In accordance with the consensus recommendations of ESPEN and EASO,^12^ the assessment of SO was divided into 3 main steps: screening, diagnosis, and staging.

Screening consisted of 2 steps. The first involved an elevated BMI or an increase in waist circumference. The second included alternative indicators or suspicion factors of low muscle function, such as clinical symptoms and general factors associated with risk (eMethods 2 in Supplement 1).^14^

The diagnostic process consisted of 2 steps: changes in skeletal muscle function parameters and subsequently changes in body composition. In this study, we used hand grip strength (HGS) to assess muscle function (eMethods 3 in Supplement 1). We used a standard cutoff of 28 kg for men and 18 kg for women.^15^ Furthermore, to explore appropriate cutoff values, we established sex-specific quintiles for HGS in this cohort (22.7 kg for men and 13.8 kg for women) as diagnostic criteria.^16^ Increased fat mass (FM) and low appendicular lean mass adjusted for body weight (ALM/W) were used as indicators of changes in body composition. FM was measured using bioelectrical impedance analysis (BIA) (eMethods 4 in Supplement 1).^17^ ALM was calculated using an equation adapted for the Chinese population, which has been widely described (eMethods 5 in Supplement 1).^18^ Cutoff values of ALM/W are 29.9% for men and 25.1% for women.^19^ We explored suitable cutoff values for body composition and used the highest 2 quintiles of FM (23.3% for men and 34% for women)^20^ and 1 SD below the sex-specific mean (31.2% for men and 24.5% for women) as cutoff values.^12^

After confirming the diagnosis of SO, the consensus recommends classifying patients with SO into stages 1 and 2 based on complications, particularly those attributable to changes in body composition and skeletal muscle function (metabolic diseases, disabilities resulting from high FM and/or low muscle mass, and cardiovascular and respiratory diseases).^12^

### Collection of Covariate Data

All patients underwent comprehensive interviews and assessments by professional nutritionists and physicians after admission. Covariates included baseline data (sex, age, education level, family history of cancer, cancer stage, diabetes, hypertension, coronary heart disease, smoking, alcohol consumption, and treatment modality), laboratory data (creatinine, albumin, and neutrophil to lymphocyte ratio [NLR]), anthropometric indicators (maximum upper arm circumference [MAC], triceps skinfold thickness [TSF], and calf circumference), and questionnaire scales (Karnofsky Performance Score [KPS], recent weight loss in the last 6 months, and nutritional support).

### Outcomes

The primary outcome of this study was overall survival (OS). OS was defined as the time from the date the patient was diagnosed with cancer to death or last follow-up (June 30, 2022). Secondary outcomes included patient QoL and risk of admission to the intensive care unit. We assessed the QoL of patients with cancer using the European Organization for Research and Treatment of Cancer Quality of Life Questionnaire (QLQ-C30) (eMethods 6 in Supplement 1).^21^

### Statistical Analysis

All statistical analyses were performed using R software, version 4.2.0 (R Project for Statistical computing). A 2-sided *P* value less than .05 was considered statistically significant. All data, normally or not normally distributed, were presented as means and SDs or medians and IQRs and compared between groups using *t* tests, analysis of variance tests, or Mann-Whitney *U* tests. Categorical variables are expressed as number and percentage and were compared between groups using the χ^2^ test. Bonferroni correction was used in the comparison of multiple groups. Univariable and multivariable logistic regression analyses were used to explore factors associated with risk for SO. Kaplan-Meier curves and log-rank test were used to illustrate survival among different groups. Cox proportional hazards models were used to investigate the association between SO and OS as described by hazard ratios (HR) and 95% CIs. Subgroup and interaction analyses were conducted to explore the association between SO and OS among different subgroups. To validate the robustness of the results, sensitivity analyses were performed (eMethods 7 in Supplement 1). Furthermore, because of the relatively low prevalence of SO, inverse probability treatment weighting (IPTW) was applied (eMethods 8 in Supplement 1), which may have helped balance the observed baseline characteristic differences between the 2 groups, thus mitigating bias due to confounding factors.^22^ Data were analyzed from June to December 2023.

## Results

### Baseline Characteristics and Factors Associated With Risk for SO

A total of 6790 participants were included in this study (eFigure 1 in Supplement 1). Their mean (SD) age was 59.64 (10.77) years, and 3489 were female (51.4%). Over a median (IQR) follow-up of 6.83 (5.67-7.04) years, 2103 patients died. Compared with patients without SO, those with SO were older, had lower education levels, and were more likely to have comorbidities. In addition, they had higher serum creatinine, MAC, TSF, and calf circumference (eTable 1 in Supplement 1). Furthermore, we categorized patients into nonobese, obese without sarcopenia, and SO or into nonsarcopenia, sarcopenia without obesity, and SO to elucidate the prevalence and characteristics of SO in patients with obesity or in patients with sarcopenia (Table 1). Bonferroni correction provided more details on intergroup comparisons (eTable 2 in Supplement 1).

**Table 1.** Baseline Characteristics

Level	Patients, No. (%)			*P* value	Patients, No. (%)			*P* value
	Nonsarcopenia (n = 5311)	Sarcopenia			Nonobesity (n = 4814)	Obesity		
		Sarcopenia without obesity (n = 1183)	SO (n = 296)			Obesity without sarcopenia (n = 1680)	SO (n = 296)	
Age, mean (SD), y	59.32 (10.89)	59.07 (9.95)	67.61 (8.68)	<.001	59.85 (10.92)	57.64 (9.96)	67.61 (8.68)	<.001
Sex								
Male	2504 (47.1)	668 (56.5)	129 (43.6)	<.001	2491 (51.7)	681 (40.5)	129 (43.6)	<.001
Female	2807 (52.9)	515 (43.5)	167 (56.4)		2323 (48.3)	999 (59.5)	167 (56.4)	
KPS, median (IQR)	90.00 (80.00-90.00)	90.00 (90.00-100.00)	90.00 (90.00-90.00)	<.001	90.00 (80.00-90.00)	90.00 (90.00-100.00)	90.00 (90.00-90.00)	<0.001
High school education and above	1972 (37.1)	513 (43.4)	87 (29.4)	<.001	1783 (37.0)	702 (41.8)	87 (29.4)	<.001
Family history of tumor	947 (17.8)	202 (17.1)	47 (15.9)	.60	809 (16.8)	340 (20.2)	47 (15.9)	.005
Diabetes	521 (9.8)	109 (9.2)	60 (20.3)	<.001	421 (8.7)	209 (12.4)	60 (20.3)	<.001
Hypertension	955 (18.0)	210 (17.8)	116 (39.2)	<.001	741 (15.4)	424 (25.2)	116 (39.2)	<.001
CHD	286 (5.4)	66 (5.6)	47 (15.9)	<.001	237 (4.9)	115 (6.8)	47 (15.9)	<.001
Chronic liver disease	98 (1.8)	16 (1.4)	4 (1.4)	.44	83 (1.7)	31 (1.8)	4 (1.4)	.83
COPD	16 (0.3)	13 (1.1)	4 (1.4)	<.001	22 (0.5)	7 (0.4)	4 (1.4)	.09
Anemia	110 (2.1)	49 (4.1)	3(1.0)	<.001	134 (2.8)	25 (1.5)	3 (1.0)	.003
Smoking	2269 (42.7)	607 (51.3)	122 (41.2)	<.001	2246 (46.7)	630 (37.5)	122 (41.2)	<.001
Alcohol use	981 (18.5)	294 (24.9)	42 (14.2)	<.001	1001 (20.8)	274 (16.3)	42 (14.2)	<.001
Stage								
1	718 (13.5)	103 (8.7)	41 (13.9)	<.001	534 (11.1)	287 (17.1)	41 (13.9)	<.001
2	1215 (22.9)	240 (20.3)	66 (22.3)		1046 (21.7)	409 (24.3)	66 (22.3)	
3	1504 (28.3)	345 (29.2)	76 (25.7)		1417 (29.4)	432 (25.7)	76 (25.7)	
4	1874 (35.3)	495 (41.8)	113 (38.2)		1817 (37.7)	552 (32.9)	113 (38.2)	
Surgery	943 (17.8)	240 (20.3)	61 (20.6)	.07	861 (17.9)	322 (19.2)	61 (20.6)	.29
Chemotherapy	3294 (62.0)	709 (59.9)	174 (58.8)	.25	2952 (61.3)	1051 (62.6)	174 (58.8)	.41
Radiotherapy	183 (3.4)	47 (4.0)	18 (6.1)	.10	174 (3.6)	56 (3.3)	18 (6.1)	.12
NLR, median (IQR)	2.27 (1.53-3.58)	2.77 (1.64-4.56)	2.56 (1.69- 3.89)	<.001	2.40 (1.56-3.92)	2.17 (1.51-3.39)	2.56 (1.69- 3.89)	<.001
SCR, median (IQR), μmol/L	61.90 (53.40-71.95)	61.70 (52.20-72.20)	64.10 (55.27-74.50)	.02	61.70 (53.10-71.77)	62.20 (53.70-72.90)	64.10 (55.27- 74.50)	.004
ALB, median (IQR), g/L	39.50 (36.10-42.50)	37.30 (33.80-40.70)	39.70 (36.10-42.02)	<.001	38.60 (35.10-41.80)	40.30 (37.10-43.20)	39.70 (36.10- 42.02)	<.001
MAC, median (IQR), cm	27.00 (25.00-29.00)	25.00 (23.00-27.00)	29.00 (27.00-31.00)	<.001	26.00 (24.00-27.50)	29.00 (27.80-31.00)	29.00 (27.00- 31.00)	<.001
TSF, median (IQR), mm	18.00 (12.45-22.00)	14.00 (10.00-20.00)	22.00 (16.00-26.00)	<.001	16.00 (12.00-20.00)	22.00 (17.00-26.00)	22.00 (16.00- 26.00)	<.001
Calf circumference, median (IQR), cm	34.00 (32.00-36.50)	32.00 (30.00-34.50)	36.00 (34.00-38.00)	<.001	33.00 (30.90-35.00)	36.50 (34.50-38.92)	36.00 (34.00- 38.00)	<.001
Weight loss	1485 (28.0)	1101 (93.1)	114 (38.5)	<.001	1999 (41.5)	587 (34.9)	114 (38.5)	<.001
ICU	859 (16.2)	209 (17.7)	58 (19.6)	.17	774 (16.1)	294 (17.5)	58 (19.6)	.15
Nutrition support	440 (8.3)	56 (4.7)	29 (9.8)	<.001	420 (8.7)	76 (4.5)	29 (9.8)	<.001
BMI, mean (SD)^a^	23.38 (3.33)	21.22 (3.22)	28.36 (2.62)	<.001	21.48 (2.33)	27.31 (2.07)	28.36 (2.62)	<.001
FM, median (IQR)	26.90 (20.40-33.50)	22.60 (16.80-29.10)	37.25 (33.45-42.42)	<.001	23.60 (17.80-29.80)	33.90 (27.70-38.20)	37.25 (33.45-42.42)	<.001
HGS, median (IQR), kg	23.80 (18.50-31.20)	22.50 (17.10-30.50)	16.15 (12.67-23.10)	<.001	23.00 (17.50-30.30)	25.00 (19.80-33.32)	16.15 (12.67-23.10)	<.001
ALM/W, median (IQR)	32.09 (26.92-34.64)	28.24 (25.76-32.84)	24.37 (23.81-30.31)	<.001	31.80 (26.56-33.87)	25.83 (24.90-30.99)	24.37 (23.81-30.31)	<.001

Abbreviations: ALB, albumin; ALM/W, appendicular lean mass adjusted for body weight; BMI, body mass index; CHD, chronic heart disease; COPD, chronic obstructive pulmonary disease; FM, fat mass; HGS, hand grip strength; ICU, intensive care unit; KPS, Karnofsky Performance Score; MAC, maximum upper arm circumference; NLR, neutrophil to lymphocyte ratio; SCR, serum creatinine; SO, sarcopenic obesity; TSF, triceps skinfold thickness.

^a^ Body mass index is calculated as weight in kilograms divided by height in meters squared.

**Figure 1.**
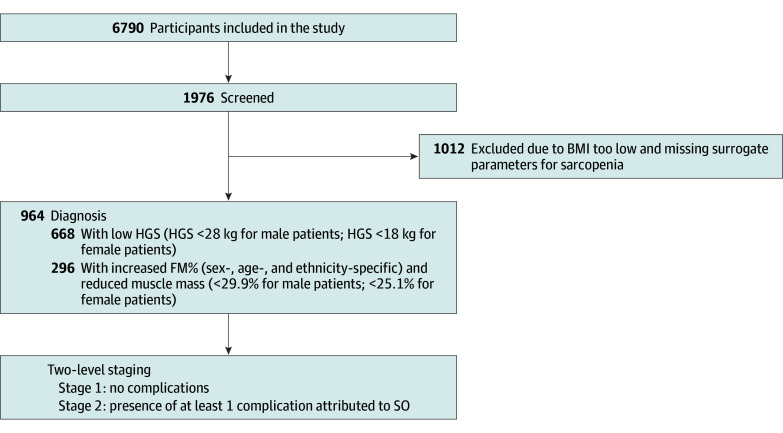
Screening, Diagnosis, and Staging Procedure for Sarcopenic Obesity (SO) BMI indicates body mass index; FM, fat mass; HGS, hand grip strength.

According to expert consensus on SO, we screened, diagnosed, and staged 6790 participants (Figure 1). Among them, there were 1976 patients with BMI 25 or higher (29.10%), 1809 patients with low HGS (26.64%), and 1621 patients with low ALM/W (23.87%). A total of 296 participants (4.36% of the total cohort and 14.98% of the obesity subgroup) were diagnosed with SO. Since patients had cancer by definition, all of them were assigned to stage 2 disease.

Subsequently, we described the prevalence of SO in different age groups, sexes, and types of cancer (eFigure 2 in Supplement 1). Regardless of sex in the overall population, the prevalence of SO gradually increased with age. Breast cancer (75 of 1263 [5.94%]), lung cancer (120 of 2558 [4.69%]), and colorectal cancer (45 of 987 [4.56%]) had the highest prevalences. Logistic regression analysis showed that sex, age, education level, smoking, advanced stage, radiotherapy, weight loss, and NLR of 3 or greater were associated with SO in patients with cancer (eTable 3 in Supplement 1).

### Association Between SO and OS

**Figure 2.**
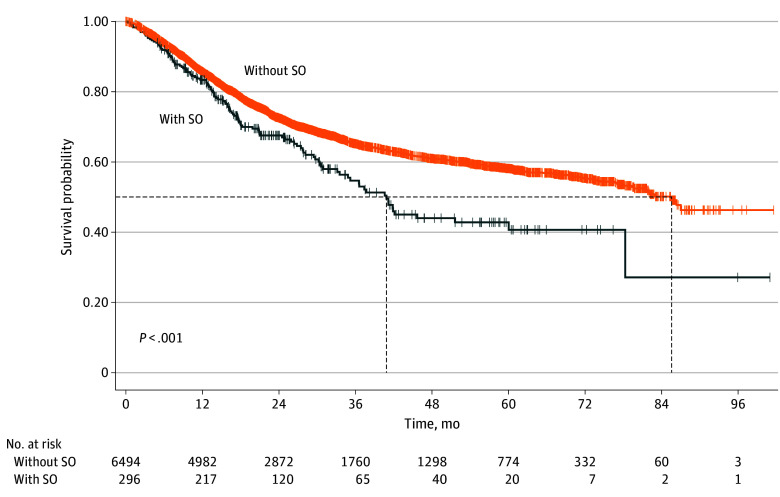
Kaplan-Meier Curves of Overall Survival for Patients Stratified by Sarcopenic Obesity (SO) Dotted lines indicate indicates censored data.

Kaplan-Meier curves indicated that patients with SO had significantly poorer OS than those without SO (χ^2^_1_ = 14.7; *P* < .001) (Figure 2). Subsequently, we investigated the association of SO with its diagnostic components and OS (Table 2). After adjusting for potential confounding factors, SO was associated with poor OS in patients with cancer (HR, 1.54; 95% CI, 1.23-1.92). This association was also observed in both men (HR, 1.51; 95% CI, 1.09-2.10) and women (HR, 1.53; 95% CI, 1.12-2.07). Regarding the SO components, only HGS showed a significant association with OS (HR, 1.15; 95% CI, 1.04-1.28).

**Table 2.** Associations Between SO and Its Components and OS

Model^a^	All		Men		Women	
	HR (95% CI)	*P* value	HR (95% CI)	*P* value	HR (95% CI)	*P* value
SO						
Model 0	1.43 (1.19-1.72)	<.001	1.38 (1.07-1.79)	.02	1.58 (1.21-2.06)	<.001
Model 1	1.35 (1.09-1.68)	.01	1.28 (0.93-1.77)	.13	1.41 (1.05-1.90)	.02
Model 2	1.54 (1.23-1.92)	<.001	1.51 (1.09-2.10)	.01	1.53 (1.12-2.07)	.01
Body mass index						
Model 0	0.83 (0.75-0.91)	<.001	0.94 (0.83-1.07)	.37	0.81 (0.70-0.94)	.004
Model 1	0.86 (0.78-0.96)	.01	0.85 (0.73-0.99)	.04	0.89 (0.76-1.04)	.13
Model 2	1.10 (0.96-1.25)	.16	1.01 (0.85-1.21)	.89	1.22 (1.01-1.48)	.04
Fat mass						
Model 0	1.19 (1.06-1.33)	.003	1.13 (0.99-1.30)	.08	1.05 (0.86-1.29)	.60
Model 1	0.94 (0.82-1.07)	.36	0.94 (0.80-1.11)	.48	0.99 (0.80-1.23)	.91
Model 2	1.09 (0.94-1.25)	.24	1.07 (0.90-1.28)	.45	1.13 (0.90-1.43)	.29
Hand grip strength						
Model 0	1.44 (1.32-1.57)	<.001	1.28 (1.14-1.43)	<.001	1.72 (1.51-1.97)	<.001
Model 1	1.32 (1.20-1.45)	<.001	1.20 (1.05-1.36)	.01	1.49 (1.29-1.73)	<.001
Model 2	1.15 (1.04-1.28)	.01	1.07 (0.93-1.23)	.37	1.25 (1.07-1.46)	.004
Appendicular lean mass adjusted for body weight						
Model 0	1.05 (0.93-1.18)	.43	0.97 (0.83-1.14)	.75	1.12 (0.94-1.35)	.21
Model 1	0.90 (0.78-1.04)	.15	0.88 (0.73-1.06)	.17	0.90 (0.72-1.12)	.34
Model 2	1.07 (0.92-1.25)	.38	1.05 (0.85-1.30)	.65	1.05 (0.83-1.32)	.68

^a^ Model 0 was the crude model. Model 1 was adjusted for age, sex, cancer type, stage, treatment. Model 2 was adjusted for age, sex, cancer type, stage, treatment, education level, neutrophil to lymphocyte ratio, family history of cancer, diabetes, hypertension, chronic heart disease, alcohol use, smoking, serum creatinine, albumin, maximum upper arm circumference, triceps skinfold thickness, calf circumference, weight loss, nutrition support, body mass index, hand grip strength, fat mass, and appendicular lean mass adjusted for body weight.

In our subgroup and interaction analyses, we did not observe any significant interactions between SO and the covariates (eFigure 3 in Supplement 1). Subsequently, we described the association between SO and outcomes in various groups of patients with cancer. Kaplan-Meier curves (eFigure 4 in Supplement 1) and multivariable Cox regression analysis (eTable 4 in Supplement 1) indicated that SO was associated with poor outcomes in patients with lung, colorectal, and pancreatic cancers. Despite our classification of cancer types, Cox regression results showed that SO was associated with worse outcomes in both digestive system cancers (HR, 1.53; 95% CI, 1.07-2.18) and nondigestive system cancers (HR, 1.58; 95% CI, 1.22-2.04).

Furthermore, we compared the differences in outcomes among the 4 groups (no sarcopenia and no obesity, no sarcopenia and obesity, sarcopenia and no obesity, and sarcopenia and obesity) (Table 3). The results showed that, regardless of sex specificity (men: HR, 1.38; 95% CI, 0.98-1.95; women: HR, 1.94; 95% CI, 1.45-2.59) or in the whole cohort (HR, 1.71; 95% CI,1.38-2.13), patients with SO had worse outcomes compared with those without SO (no sarcopenia and no obesity).

**Table 3.** The Association Between Sarcopenia, Obesity, and Outcomes Stratified by Sex

Model^a^	No sarcopenia and no obesity	No sarcopenia and obesity	*P* value	Sarcopenia and no obesity	*P* value	Sarcopenic obesity	*P* value
All							
Model 0	1 [Reference]	0.67 (0.60-0.76)	<.001	1.15 (1.02-1.44)	.04	1.37 (1.12-1.68)	.002
Model 1	1 [Reference]	0.77 (0.68-0.87)	<.001	1.29 (1.02-1.63)	.03	1.38 (1.13-1.70)	.002
Model 2	1 [Reference]	0.98 (0.85-1.13)	.80	1.31 (1.03-1.67)	.03	1.71 (1.38-2.13)	<.001
Men							
Model 0	1 [Reference]	0.76 (0.64-0.89)	<.001	0.81 (0.44-1.52)	.52	1.29 (0.93-1.80)	.13
Model 1	1 [Reference]	0.79 (0.67-0.93)	.005	1.12 (0.60-2.11)	.72	1.15 (0.82-1.61)	.41
Model 2	1 [Reference]	0.93 (0.77-1.13)	.49	1.11 (0.58-2.11)	.75	1.38 (0.98-1.95)	.07
Women							
Model 0	1 [Reference]	0.68 (0.57-0.82)	<.001	1.63 (1.27-2.09)	<.001	1.74 (1.34-2.25)	<.001
Model 1	1 [Reference]	0.75 (0.63-0.90)	.002	1.25 (1.06-1.63)	.02	1.55 (1.18-2.02)	.001
Model 2	1 [Reference]	1.06 (0.86-1.32)	.59	1.27 (0.96-1.66)	.09	1.94 (1.45-2.59)	<.001

^a^ Model was adjusted for age, sex, cancer type, stage, treatment, education level, neutrophil to lymphocyte ratio, family history of cancer, diabetes, hypertension, chronic heart disease, alcohol use, smoking, serum creatinine, albumin, maximum upper arm circumference, triceps skinfold thickness, calf circumference, weight loss, nutrition support, body mass index, hand grip strength, fat mass, and appendicular lean mass adjusted for body weight.

### Additional Analyses

Several sensitivity analyses were conducted (eTable 5 in Supplement 1). Considering that the impact of age on survival may not be linear, we also included the squared age as a covariate, and patients with SO also had worse outcomes (HR, 1.74; 95% CI, 1.43-2.12). To avoid the influence of treatment, we divided patients into newly diagnosed and treated. The results suggest that regardless of whether patients had received treatment, SO remained associated with poorer outcomes (eFigure 5 in Supplement 1). Then we performed IPTW analysis and found no significant difference in age, sex, and stage of matched patients (eTable 6 in Supplement 1). The IPTW analysis suggested that even after assigning the appropriate weights to each patient, patients with cancer with SO still had poorer outcomes (HR, 2.00; 95% CI, 1.42-2.82) (eFigure 6 and eTable 7 in Supplement 1). We also explored the association between SO and other outcomes. Regarding QoL (eTable 8 in Supplement 1), patients with SO had worse mean (SD) global health status score (70.28 [21.58] vs 66.54 [21.57]; *P* = .02) and physical (85.86 [18.96] vs 81.34 [20.58]; *P* < .001), role (85.10 [23.27] vs 80.58 [23.84]; *P* = .01), emotional (89.81 [16.07] vs 87.07 [18.29]; *P* = .02), and cognitive functioning (89.43 [16.04] VS. 86.17 [18.72]; *P* = .01) scores. In terms of symptoms, patients with SO were more likely to experience fatigue (17.90 [21.12] vs 21.42 [22.58]; *P* = .02) and dyspnea (10.92 [19.94] vs 14.78 [23.49]; *P* = .01). Regarding admission rates to the ICU (eTable 9 in Supplement 1), SO was associated with a higher admission rate (odds ratio [OR] before IPTW, 2.39; 95% CI, 1.06-5.29; OR after IPTW, 2.55; 95% CI, 1.18-5.41).

### Exploring the Optimal Cutoff Values

As mentioned in the Methods, we explored the cohort-specific cutoff values for SO diagnosis (eFigure 7 in Supplement 1). Among the 1976 patients screened according to BMI and symptoms, 298 patients were identified as having the lowest HGS. After assessing body composition, 140 patients (2.06%) were diagnosed with SO, all of whom were classified as stage 2. When stratified by age, the prevalence of SO increased (eFigure 8 in Supplement 1). Cox regression analysis (eTable 10 in Supplement 1) indicated that, despite using a more stringent diagnosis approach, SO remained associated with poor outcomes in patients with cancer (HR, 1.98; 95% CI, 1.49-2.64).

## Discussion

This study explored the prevalence of SO diagnosed using the ESPEN and EASO criteria and their association with outcomes in a cohort of 6790 patients with cancer. Results revealed that 4.36% of the whole cohort had SO, which increased to 14.98% when considering the obesity subgroup. SO prevalence increased with age. In terms of cancer type, SO was more prevalent in patients with breast, lung, and colorectal cancers.

Although the definitions of sarcopenia and obesity are well established, the definition of SO has been a subject of controversy, leading to substantial variability in reported SO prevalence based on selected definitions and diagnostic criteria.^23^ A study^24^ showed a high prevalence of SO in the elderly population, with the prevalence ranging from 1.3% to 12.5% depending on the criteria. Another study^25^ used 8 different methods to define SO, resulting in prevalence rates ranging from 3.6% to 94%. The lack of standardization in SO definitions prompted the SO initiative by ESPEN and EASO. This guideline aimed to be independent of age and disease. In early studies implementing the ESPEN-EASO algorithm, prevalence of SO was 7.9% in patients with stroke and was negatively correlated with improvements in daily living activities.^26^ The prevalence of ESPEN-EASO–defined SO was reported to be 4.5% to 10% in free-living older adults using dual-energy x-ray absorptiometry or BIA to assess muscle mass.^27,28,29^ In contrast, the population in this study is patients with cancer; therefore, metabolic complications are expected to be common, with particular regard to high systemic inflammation and metabolic rate, which may contribute to skeletal muscle wasting and loss of fat mass.^30^ Patients with cancer are often exposed to acute catabolic events due to complications and treatments such as chemotherapy and radiation therapy, which may also reduce food intake.^31,32,33^ Nutritional complications with concomitant long-term loss of weight could have potentially limited the prevalence of SO in the whole cohort. However, the prevalence of SO among patients with obesity was high at nearly 15%, suggesting that this remains a noteworthy issue. The finding of high prevalence of SO in patients with breast cancer is intriguing, since breast cancer is typically less catabolic than other forms of cancer. However, obesity is a specific risk factor for breast cancer.^34^ It is possible that obesity duration in patients with breast cancer was longer than others, with more profound metabolic derangements with negative impact on skeletal muscle.

Previous studies have explored the relationship between SO and clinical outcomes in various disease conditions using different definitions.^35^ Lack of consensus has continued to limit implementation of SO diagnosis in clinical practice. In this study, Cox regression analysis demonstrated that ESPEN-EASO–defined SO was significantly associated with poor outcomes, particularly in patients with lung, colorectal, and pancreatic cancers. Secondary outcome analyses revealed associations among SO, QoL, and risk of admission to the ICU of patients with cancers. Based on the current findings, SO diagnosis using the ESPEN-EASO algorithm appears to enhance the ability to identify patients at higher risk of adverse outcomes. It is also important to point out that low HGS is the main component leading to the diagnosis of SO and was independently associated with lower OS, whereas the association with muscle mass was not observed in this study. These findings are consistent with recent studies, where low muscle function was the most prominent derangement.^27,28^ The role of HGS has been extensively discussed. It is not only associated with the occurrence of several cancers, such as liver cancer and breast cancer,^36^ but also affects the outcomes of patients with cancer.^37,38^ As for skeletal muscle mass, although computed tomography–assessed low muscle mass has been reported to predict reduced survival in patients with cancer with obesity,^39^ this association has not been invariably confirmed in all subsequent reports.^40,41,42^ Variability in cancer type, clinical conditions, and complications could contribute to some discrepancies. In addition, methods for muscle mass assessment could also affect results. BIA may have limitations due to its inability to detect altered muscle composition and potential fat infiltration which may be particularly relevant in the presence of obesity.^43^ For patients with concurrent obesity and sarcopenia in chronic disease, the degree of intermuscular fat infiltration as determined by CT has been reported to be significantly increased and closely associated with outcomes.^44^ The use of BIA in this study may therefore have overestimated the functional muscle mass in some patients. For certain populations and specific conditions, BIA may still have limitations compared with CT.^43^ Standardizing HGS and body composition assessment techniques may be required to optimize effectiveness of the ESPEN-EASO algorithm in the future. For regions with poorer economic development or inadequate health care, increasing the emphasis on HGS in the diagnosis of SO might be worth exploring.

Since the ESPEN-EASO algorithm envisions the potential use of optimized cutoff values to detect and diagnose SO in different populations and cohorts, we explored a method for selecting additional cutoff values based on previous studies.^16,19,45^ After adopting stricter criteria, the rate of SO decreased to 2.06%, despite the poorer outcomes of patients with SO. Therefore, it is important to note that overly strict criteria may reduce the sensitivity of SO diagnosis in patients with cancer.

### Limitations

This study had several limitations. First, the study population is Chinese patients with cancer, and the racial specificity of body composition cannot be ignored. The generalizability of these results may be limited. Second, although this study validated and explored the diagnosis of SO using different criteria, we may not have identified the optimal cutoff values to maximize the detection rate of SO. Third, the use of BIA for muscle mass assessment may not be accurate for older patients and those with a high BMI. Precise imaging data are necessary as they can more accurately identify intermuscular fat mass. Fourth, many patients in advanced stages may be bedridden for extended periods due to frailty or other complications, making it difficult to cooperate with muscle function or body composition measurements.

## Conclusions

In patients with solid tumors, the prevalence of SO, as defined by the ESPEN-EASO criteria, was 4.36% in the whole cohort and 14.98% in the obesity subgroup. Higher rates were observed in patients with breast, lung and colorectal cancers. SO was significantly associated with QoL, ICU admission rate, and OS in patients with cancer. Patients with cancer should undergo regular screening and diagnosis for SO, and the ESPEN-EASO algorithm may be an effective tool for the clinical implementation of SO.

## Notes

Supplement 1.
eMethods 1.

eMethods 2.

eMethods 3.

eMethods 4.

eMethods 5.

eMethods 6.

eMethods 7.

eMethods 8.

eReferences.
**eFigure 1.** Flow Chart **eFigure 2. **Prevalence Rate of SO in Patients of Different Ages and Cancer Types **eFigure 3.** Subgroup Analysis **eFigure 4. **Kaplan-Meier Curves of OS for Patients Stratified by SO Among Patients With Common Cancer Types **eFigure 5.** Kaplan-Meier Curves of SO and OS in Newly Diagnosed and Treated Patients With Cancer **eFigure 6.** Kaplan-Meier Curves of OS for Patients Stratified by SO After IPTW **eFigure 7. **Screening, Diagnosis, and Staging Procedure for SO via Cohort-Specific Cutoff **eFigure 8.** Prevalence Rate of SO in Patients of Different Ages and Cancer Types (Cohort-Specific Cutoff) **eTable 1.** Baseline Characteristics Stratified by SO **eTable 2.** *P* Value for Bonferroni Correction **eTable 3.** Univariate and Multivariate Logistic Regression for Analyzing Associated Factors for SO **eTable 4.** The Association Between SO and Outcomes Stratified by Cancer Types and Sex **eTable 5.** Sensitivity Analyses **eTable 6.** Baseline Characteristics After IPTW **eTable 7. **Associations Between SO and OS After IPTW **eTable 8. **QLQc30 for Patients With SO and Patients Without SO **eTable 9. **Association Between SO and Risk of ICU Admission **eTable 10. **Associations Between SO (Cohort-Specific Cutoff) and its Components and OS

Supplement 2.
Data Sharing Statement

## References

[1] Shimizu A, Maeda K, Ueshima J, . Prevalence of sarcopenic obesity based on newly proposed diagnostic criteria and functional outcomes in older adults undergoing rehabilitation. Mech Ageing Dev. 2022;208:111728. doi:10.1016/j.mad.2022.111728 36084796

[2] Cruz-Jentoft AJ, Bahat G, Bauer J, ; Writing Group for the European Working Group on Sarcopenia in Older People 2 (EWGSOP2), and the Extended Group for EWGSOP2. Sarcopenia: revised European consensus on definition and diagnosis. Age Ageing. 2019;48(4):601. doi:10.1093/ageing/afz046 31081853 PMC6593317

[3] Kirk B, Cawthon PM, Arai H, ; Global Leadership Initiative in Sarcopenia (GLIS) group. The conceptual definition of sarcopenia: Delphi consensus from the Global Leadership Initiative in Sarcopenia (GLIS). Age Ageing. 2024;53(3):afae052. doi:10.1093/ageing/afae052 38520141 PMC10960072

[4] Feliciano EMC, Kroenke CH, Meyerhardt JA, . Association of systemic inflammation and sarcopenia with survival in nonmetastatic colorectal cancer: results from the C SCANS study. JAMA Oncol. 2017;3(12):e172319. doi:10.1001/jamaoncol.2017.2319 28796857 PMC5824285

[5] Hu J, Yang J, Yu H, . Effect of sarcopenia on survival and health-related quality of life in patients with hepatocellular carcinoma after hepatectomy. Cancers (Basel). 2022;14(24):6144. doi:10.3390/cancers14246144 36551629 PMC9776353

[6] Feng Y, Wang L, Guo F, . Predictive impact of sarcopenia in advanced non-small cell lung cancer patients treated with immune checkpoint inhibitors: a retrospective study. Heliyon. 2024;10(5):e27282. doi:10.1016/j.heliyon.2024.e27282 38463845 PMC10923705

[7] Meza-Valderrama D, Marco E, Dávalos-Yerovi V, . Sarcopenia, malnutrition, and cachexia: adapting definitions and terminology of nutritional disorders in older people with cancer. Nutrients. 2021;13(3):761. doi:10.3390/nu13030761 33652812 PMC7996854

[8] Avgerinos KI, Spyrou N, Mantzoros CS, Dalamaga M. Obesity and cancer risk: emerging biological mechanisms and perspectives. Metabolism. 2019;92:121-135. doi:10.1016/j.metabol.2018.11.001 30445141

[9] Ge YZ, Liu T, Deng L, ; Investigation on the Nutrition Status and Clinical Outcome of Common Cancers (INSCOC) Group. The age-related obesity paradigm: results from two large prospective cohort studies. J Cachexia Sarcopenia Muscle. 2024;15(1):442-452. doi:10.1002/jcsm.13415 38146198 PMC10834317

[10] Cespedes Feliciano EM, Kroenke CH, Caan BJ. The plausibility of the obesity paradox in cancer-response-reply to point. Cancer Res. 2018;78(8):1904-1905. doi:10.1158/0008-5472.CAN-17-3590 29654152 PMC5975254

[11] Baumgartner RN, Wayne SJ, Waters DL, Janssen I, Gallagher D, Morley JE. Sarcopenic obesity predicts instrumental activities of daily living disability in the elderly. Obes Res. 2004;12(12):1995-2004. doi:10.1038/oby.2004.250 15687401

[12] Donini LM, Busetto L, Bischoff SC, . Definition and diagnostic criteria for sarcopenic obesity: ESPEN and EASO consensus statement. Clin Nutr. 2022;41(4):990-1000. doi:10.1016/j.clnu.2021.11.014 35227529

[13] Xu H, Song C, Yin L, . Extension protocol for the Investigation on Nutrition Status and Clinical Outcome of Patients with Common Cancers in China (INSCOC) study: 2021 update. Precis Nutr. 2022;1:e00014. doi:10.1097/PN9.0000000000000014

[14] Batsis JA, Mackenzie TA, Bartels SJ, Sahakyan KR, Somers VK, Lopez-Jimenez F. Diagnostic accuracy of body mass index to identify obesity in older adults: NHANES 1999-2004. Int J Obes (Lond). 2016;40(5):761-767. doi:10.1038/ijo.2015.243 26620887 PMC4854777

[15] Chen LK, Woo J, Assantachai P, . Asian working group for sarcopenia: 2019 Consensus update on sarcopenia diagnosis and treatment. J Am Med Dir Assoc. 2020;21(3):300-307.e2. doi:10.1016/j.jamda.2019.12.012 32033882

[16] Spruit MA, Sillen MJ, Groenen MT, Wouters EF, Franssen FM. New normative values for handgrip strength: results from the UK Biobank. J Am Med Dir Assoc. 2013;14(10):775.e5-775.e11. doi:10.1016/j.jamda.2013.06.013 23958225

[17] Gallagher D, Heymsfield SB, Heo M, Jebb SA, Murgatroyd PR, Sakamoto Y. Healthy percentage body fat ranges: an approach for developing guidelines based on body mass index. Am J Clin Nutr. 2000;72(3):694-701. doi:10.1093/ajcn/72.3.694 10966886

[18] Ruan GT, Ge YZ, Xie HL, . Association between systemic inflammation and malnutrition with survival in patients with cancer sarcopenia-A prospective multicenter study. Front Nutr. 2022;8:811288. doi:10.3389/fnut.2021.811288 35198586 PMC8859438

[19] Lim S, Kim JH, Yoon JW, . Sarcopenic obesity: prevalence and association with metabolic syndrome in the Korean Longitudinal Study on Health and Aging (KLoSHA). Diabetes Care. 2010;33(7):1652-1654. doi:10.2337/dc10-0107 20460442 PMC2890376

[20] Lee J, Hong YP, Shin HJ, Lee W. Associations of sarcopenia and sarcopenic obesity with metabolic syndrome considering both muscle mass and muscle strength. J Prev Med Public Health. 2016;49(1):35-44. doi:10.3961/jpmph.15.055 26841883 PMC4750513

[21] Nolte S, Liegl G, Petersen MA, ; EORTC Quality of Life Group. General population normative data for the EORTC QLQ-C30 health-related quality of life questionnaire based on 15,386 persons across 13 European countries, Canada and the Unites States. Eur J Cancer. 2019;107:153-163. doi:10.1016/j.ejca.2018.11.024 30576971

[22] Chesnaye NC, Stel VS, Tripepi G, . An introduction to inverse probability of treatment weighting in observational research. Clin Kidney J. 2021;15(1):14-20. doi:10.1093/ckj/sfab158 35035932 PMC8757413

[23] Donini LM, Busetto L, Bauer JM, . Critical appraisal of definitions and diagnostic criteria for sarcopenic obesity based on a systematic review. Clin Nutr. 2020;39(8):2368-2388. doi:10.1016/j.clnu.2019.11.024 31813698

[24] Kim TN, Yang SJ, Yoo HJ, . Prevalence of sarcopenia and sarcopenic obesity in Korean adults: the Korean sarcopenic obesity study. Int J Obes (Lond). 2009;33(8):885-892. doi:10.1038/ijo.2009.130 19564878

[25] Batsis JA, Barre LK, Mackenzie TA, Pratt SI, Lopez-Jimenez F, Bartels SJ. Variation in the prevalence of sarcopenia and sarcopenic obesity in older adults associated with different research definitions: dual-energy X-ray absorptiometry data from the National Health and Nutrition Examination Survey 1999-2004. J Am Geriatr Soc. 2013;61(6):974-980. doi:10.1111/jgs.12260 23647372

[26] Yoshimura Y, Wakabayashi H, Nagano F, . The applicability of the ESPEN and EASO-defined diagnostic criteria for sarcopenic obesity in Japanese patients after stroke: prevalence and association with outcomes. Nutrients. 2022;14(19):4205. doi:10.3390/nu14194205 36235857 PMC9570818

[27] Gortan Cappellari G, Semolic A, Zanetti M, . Sarcopenic obesity in free-living older adults detected by the ESPEN-EASO consensus diagnostic algorithm: validation in an Italian cohort and predictive value of insulin resistance and altered plasma ghrelin profile. Metabolism. 2023;145:155595. doi:10.1016/j.metabol.2023.155595 37245728

[28] Scott D, Blyth F, Naganathan V, . Sarcopenia prevalence and functional outcomes in older men with obesity: comparing the use of the EWGSOP2 sarcopenia versus ESPEN-EASO sarcopenic obesity consensus definitions. Clin Nutr. 2023;42(9):1610-1618. doi:10.1016/j.clnu.2023.07.014 37481869

[29] Schluessel S, Huemer MT, Peters A, Drey M, Thorand B. Sarcopenic obesity using the ESPEN and EASO consensus statement criteria of 2022—results from the German KORA-Age study. Obes Res Clin Pract. 2023;17(4):349-352. doi:10.1016/j.orcp.2023.08.002 37633820

[30] Setiawan T, Sari IN, Wijaya YT, . Cancer cachexia: molecular mechanisms and treatment strategies. J Hematol Oncol. 2023;16(1):54. doi:10.1186/s13045-023-01454-0 37217930 PMC10204324

[31] Johannes CM, Musser ML. Anorexia and the cancer patient. Vet Clin North Am Small Anim Pract. 2019;49(5):837-854. doi:10.1016/j.cvsm.2019.04.008 31176457

[32] Liu CA, Liu T, Li HC, . Nutrition impact symptoms: noteworthy prognostic indicators for lung cancer. Clin Nutr. 2023;42(4):550-558. doi:10.1016/j.clnu.2023.02.021 36863291

[33] Che X, Gross SM, Wang G, . Impact of consuming a Mediterranean-style diet during pregnancy on neurodevelopmental disabilities in offspring: results from the Boston Birth Cohort. Precis Nutr. 2023;2(3):e00047. doi:10.1097/PN9.0000000000000047 37744413 PMC10513021

[34] Picon-Ruiz M, Morata-Tarifa C, Valle-Goffin JJ, Friedman ER, Slingerland JM. Obesity and adverse breast cancer risk and outcome: mechanistic insights and strategies for intervention. CA Cancer J Clin. 2017;67(5):378-397. doi:10.3322/caac.21405 28763097 PMC5591063

[35] Prado CM, Lieffers JR, McCargar LJ, . Prevalence and clinical implications of sarcopenic obesity in patients with solid tumours of the respiratory and gastrointestinal tracts: a population-based study. Lancet Oncol. 2008;9(7):629-635. doi:10.1016/S1470-2045(08)70153-0 18539529

[36] Parra-Soto S, Pell JP, Celis-Morales C, Ho FK. Absolute and relative grip strength as predictors of cancer: prospective cohort study of 445 552 participants in UK Biobank. J Cachexia Sarcopenia Muscle. 2022;13(1):325-332. doi:10.1002/jcsm.12863 34953058 PMC8818619

[37] Song M, Zhang Q, Tang M, . Associations of low hand grip strength with 1 year mortality of cancer cachexia: a multicentre observational study. J Cachexia Sarcopenia Muscle. 2021;12(6):1489-1500. doi:10.1002/jcsm.12778 34545711 PMC8718026

[38] Hadzibegovic S, Porthun J, Lena A, . Hand grip strength in patients with advanced cancer: a prospective study. J Cachexia Sarcopenia Muscle. 2023;14(4):1682-1694. doi:10.1002/jcsm.13248 37318103 PMC10401539

[39] Şahin MEH, Arkbaş F, Yardimci AH, Şahin E. The effect of sarcopenia and sarcopenic obesity on survival in gastric cancer. BMC Cancer. 2023;23(1):911. doi:10.1186/s12885-023-11423-y 37770828 PMC10537530

[40] Nakayama N, Nakayama K, Nakamura K, Razia S, Kyo S. Sarcopenic factors may have no impact on outcomes in ovarian cancer patients. Diagnostics (Basel). 2019;9(4):206. doi:10.3390/diagnostics9040206 31795173 PMC6963637

[41] Maddalena C, Ponsiglione A, Camera L, . Prognostic role of sarcopenia in metastatic colorectal cancer patients during first-line chemotherapy: a retrospective study. World J Clin Oncol. 2021;12(5):355-366. doi:10.5306/wjco.v12.i5.355 34131567 PMC8173330

[42] Salati V, Mandralis K, Becce F, . Preoperative CT-based skeletal muscle mass depletion and outcomes after total laryngectomy. Cancers (Basel). 2023;15(14):3538. doi:10.3390/cancers15143538 37509201 PMC10377557

[43] Lemos T, Gallagher D. Current body composition measurement techniques. Curr Opin Endocrinol Diabetes Obes. 2017;24(5):310-314. doi:10.1097/MED.0000000000000360 28696961 PMC5771660

[44] Sabatino A, Avesani CM, Regolisti G, . Sarcopenic obesity and its relation with muscle quality and mortality in patients on chronic hemodialysis. Clin Nutr. 2023;42(8):1359-1368. doi:10.1016/j.clnu.2023.06.032 37418843

[45] Pedrero-Chamizo R, Gómez-Cabello A, Meléndez A, . Higher levels of physical fitness are associated with a reduced risk of suffering sarcopenic obesity and better perceived health among the elderly: the EXERNET multi-center study. J Nutr Health Aging. 2015;19(2):211-217. doi:10.1007/s12603-014-0530-425651448

