# Supplementary material for: Sarcopenic Obesity and Outcomes for Patients With Cancer
Source: JAMA Netw Open. 2024 Jun 14;7(6):e2417115. doi: 10.1001/jamanetworkopen.2024.17115 (PMC11179127; doi:10.1001/jamanetworkopen.2024.17115)
Supplement: Supplement 1. — eMethods 1. eMethods 2. eMethods 3. eMethods 4. eMethods 5. eMethods 6. eMethods 7. eMethods 8. eReferences. eFigure 1. Flow Chart eFigure 2. Prevalence Rate of SO in Patients of Different Ages and Cancer Types eFigure 3. Subgroup Analysis eFigure 4. Kaplan-Meier Curves of OS for Patients Stratified by SO Among Patients With Common Cancer Types eFigure 5. Kaplan-Meier Curves of SO and OS in Newly Diagnosed and Treated Patients With Cancer eFigure 6. Kaplan-Meier Curves of OS for Patients Stratified by SO After IPTW eFigure 7. Screening, Diagnosis, and Staging Procedure for SO via Cohort-Specific Cutoff eFigure 8. Prevalence Rate of SO in Patients of Different Ages and Cancer Types (Cohort-Specific Cutoff) eTable 1. Baseline Characteristics Stratified by SO eTable 2. P Value for Bonferroni Correction eTable 3. Univariate and Multivariate Logistic Regression for Analyzing Associated Factors for SO eTable 4. The Association Between SO and Outcomes Stratified by Cancer Types and Sex eTable 5. Sensitivity Analyses eTable 6. Baseline Characteristics After IPTW eTable 7. Associations Between SO and OS After IPTW eTable 8. QLQc30 for Patients With SO and Patients Without SO eTable 9. Association Between SO and Risk of ICU Admission eTable 10. Associations Between SO (Cohort-Specific Cutoff) and its Components and OS [file jamanetwopen-e2417115-s001.pdf]

Supplemental Online Content

Liu C, Liu T, Deng L, et al. Sarcopenic obesity and outcomes for patients with cancer. *JAMA Netw Open*. 2024;7(6):e2417115.
doi:10.1001/jamanetworkopen.2024.17115

eMethods 1.
eMethods 2.
eMethods 3.
eMethods 4.
eMethods 5.
eMethods 6.
eMethods 7.
eMethods 8.
eReferences.
eFigure 1. Flow Chart
eFigure 2. Prevalence Rate of SO in Patients of Different Ages and Cancer Types
eFigure 3. Subgroup Analysis
eFigure 4. Kaplan-Meier Curves of OS for Patients Stratified by SO Among Patients With Common Cancer Types
eFigure 5. Kaplan-Meier Curves of SO and OS in Newly Diagnosed and Treated Patients With Cancer
eFigure 6. Kaplan-Meier Curves of OS for Patients Stratified by SO After IPTW
eFigure 7. Screening, Diagnosis, and Staging Procedure for SO via Cohort-Specific Cutoff
eFigure 8. Prevalence Rate of SO in Patients of Different Ages and Cancer Types (Cohort-Specific Cutoff)
eTable 1. Baseline Characteristics Stratified by SO
eTable 2. *P* Value for Bonferroni Correction
eTable 3. Univariate and Multivariate Logistic Regression for Analyzing Associated Factors for SO
eTable 4. The Association Between SO and Outcomes Stratified by Cancer Types and Sex
eTable 5. Sensitivity Analyses
eTable 6. Baseline Characteristics After IPTW
eTable 7. Associations Between SO and OS After IPTW
eTable 8. QLQc30 for Patients With SO and Patients Without SO
eTable 9. Association Between SO and Risk of ICU Admission
eTable 10. Associations Between SO (Cohort-Specific Cutoff) and its Components and OS

This supplemental material has been provided by the authors to give readers additional information about their work.

© 2024 Liu C et al. *JAMA Network Open.*

eMethods 1.
Inclusion criteria: (1) patients pathologically diagnosed with solid tumors, including lung cancer, digestive system cancers (gastric cancer, liver cancer, colorectal cancer, esophageal cancer, pancreatic cancer, biliary tract cancers), female reproductive system cancers (cervical cancer, endometrial cancer, ovarian cancer), breast cancer, nasopharyngeal carcinoma, prostate cancer, bladder cancer, and brain tumors; (2) age 18 years or older; (3) patients who underwent detailed body composition measurements. The exclusion criteria were as follows: (1) patients with hematological malignancies, including leukemia, lymphoma, and myeloma; (2) patients with severe comorbidities, acute infections, or pregnancy; (3) patients lacking covariate data; and (4) patients lacking survival or QoL data.
Based on previous study, we have also defined obesity-related and non-obesity-related cancers[1]. Obesity-related cancers was defined as esophagus, stomach, colon, rectum, liver, pancreas, lung, breast, corpus uteri, ovaries, prostate, kidney, bladder, brain cancer, and others are non-obesity-related cancers.

eMethods 2.
Clinical Symptoms or Suspicion Factors for the screening of sarcopenic obesity:
(1) Age >70 yr; (2) Chronic Disease Diagnosiss (e.g. inflammatory diseases and organ failure or chronic disease) ; (3) Recent acute disease/nutritional events; (4) History-complaint of repeated falls, weakness, exhaustion, fatigability, and perceived progressive movement limitations [2].

eMethods 3.
Well-trained professionals use a handheld force gauge (EH101 model from Guangdong, China) based on strain gauge sensors to measure the hand grip strength (HGS) of patients, with a measurement accuracy of 0.1 kg. During the measurement process, patients are instructed to sit upright, place their arms on the armrest, and bend their elbows at a 90-degree angle. They are instructed to squeeze the dynamometer handle with as much force as possible within 3 seconds, measuring HGS three times and taking the average measurement results for analysis.

eMethods 4.
Using Multifrequency Radioimmunoassay (InBody S10, Beijing, China) to estimate body composition. This analysis was conducted while the patient was lying down, with two electrodes connected to each limb for each foot and hand. All procedures are carried out according to the manufacturer's recommendations.
Cut-off for FM%: 20-39y: >40% for F, >28% for M (Asians); 40-59y: >41% for F, >29% for M (Asians); 60-79y: >41% for F, >29% for M (Asians) [3].

eMethods 5.
ALM = 0.193 × body weight (kg) + 0.107 × height (cm)−4.157 × sex−0.037 × age−2.631. Bodyweight, height, and age were measured in kg, cm, and years, respectively. Male sex was coded as 1 and female sex as 2. The ALM equation model is in good agreement with double X-ray absorptiometer measurements (adjusted R2 = 0.90, standard error of estimate = 1.63 kg)[4].

eMethods 6.
QLQ-C30: The EORTC QLQ-C30 (version 3.0) was used to assess QoL.3 This 30-item, cancer-specific questionnaire includes 5 functional scales (physical, emotional, cognitive, social, and role), 3 symptom scales (fatigue, pain, and nausea/vomiting), a global health/QoL scale, and 6 single items (dyspnea, insomnia, appetite loss, constipation, diarrhea, and financial impact of disease). The 28 items measuring functional and symptom scales use a numeric scale for scores of 1 (not at all), 2 (a little), 3 (quite a bit), and 4 (very much). The 2 items concerning global QoL use a scale from 1 (very poor) to 7 (excellent). The raw scores were linearly transformed to give standard scores in the range of 0 to 100 for each of the scales and single items, as described by the EORTC[5].

© 2024 Liu C et al. *JAMA Network Open.*

eMethods 7.

Sensitivity analysis included four parts. First, we excluded patients with less than one year of follow-up to avoid the phenomenon of reverse causation due to early occurrence of outcomes; second, we excluded patients with a high BMI, as the measurement of body composition was done using BIA, and a high BMI might affect the calculation of body composition; third,considering that the impact of age on survival may not be linear, we also included the squared age as one of the covariates. Finally, considering that this is a longitudinal cohort, in order to avoid the influence of time on factors, we also corrected time-varying covariants.

eMethods 8.

Inverse probability treatment weighting (IPTW): IPTW is a form of propensity score weighting. Due to the low incidence rate, the standardized mean difference between the two groups of patients is large. In order to further compensate for potential bias caused by baseline differences, we conducted IPTW. Its principle is to take the reciprocal of the probability of each observation in the original data as the weight of the observed value, correcting the estimation bias caused by missing data or biased sampling[6]. Simply put, weights are used to shrink individuals who are oversampled. The purpose of weighting is to eliminate selected differences. Suppose the probability of being selected for individual A is e, then we assign a weight of 1/e to them, meaning that the smaller the probability of individual A being selected, the greater their weight in the sample. Therefore, in this study, the incidence rate of SO is low, and the probability of being selected is small. In the sample after IPTW, the number of people with SO will increase due to the large weights assigned to them, meaning that one SO patient may represent 10-20 people.

© 2024 Liu C et al. *JAMA Network Open.*

eReferences

[1] He Q, Xia B, Liu A, Li M, Zhou Z, Cheung EC, Kuo ZC, Wang B, Li F, Tang Y, Zheng Z, Sun R, Hu YJ, Meng W, He Y, Yuan J, Zhang C. Association of body composition with risk of overall and site-specific cancers: A population-based prospective cohort study. Int J Cancer. 2021 Oct 1;149(7):1435-1447. doi: 10.1002/ijc.33697. Epub 2021 Jun 4. PMID: 34019699.

[2] Donini LM, Busetto L, Bischoff SC, et al. Definition and diagnostic criteria for sarcopenic obesity: ESPEN and EASO consensus statement. Clin Nutr. 2022 Apr;41(4):990-1000. doi: 10.1016/j.clnu.2021.11.014. Epub 2022 Feb 22. PMID: 35227529.

[3] Gallagher D, Heymsfield SB, Heo M, et al. Healthy percentage body fat ranges: an approach for developing guidelines based on body mass index. Am J Clin Nutr. 2000 Sep;72(3):694-701. doi: 10.1093/ajcn/72.3.694. PMID: 10966886.

[4] Wen X, Wang M, Jiang CM, Zhang YM. Anthropometric equation for estimation of appendicular skeletal muscle mass in Chinese adults. Asia Pac J Clin Nutr. 2011;20(4):551-6. PMID: 22094840.

[5] Nolte S, Liegl G, Petersen MA, et al; EORTC Quality of Life Group. General population normative data for the EORTC QLQ-C30 health-related quality of life questionnaire based on 15,386 persons across 13 European countries, Canada and the Unites States. Eur J Cancer. 2019 Jan;107:153-163. doi: 10.1016/j.ejca.2018.11.024. Epub 2018 Dec 19. PMID: 30576971.

[6] Austin PC, Stuart EA. Moving towards best practice when using inverse probability of treatment weighting (IPTW) using the propensity score to estimate causal treatment effects in observational studies. Stat Med. 2015 Dec 10;34(28):3661-79. doi: 10.1002/sim.6607. Epub 2015 Aug 3. PMID: 26238958; PMCID: PMC4626409.

© 2024 Liu C et al. *JAMA Network Open.*

eFigure 1. Flow Chart

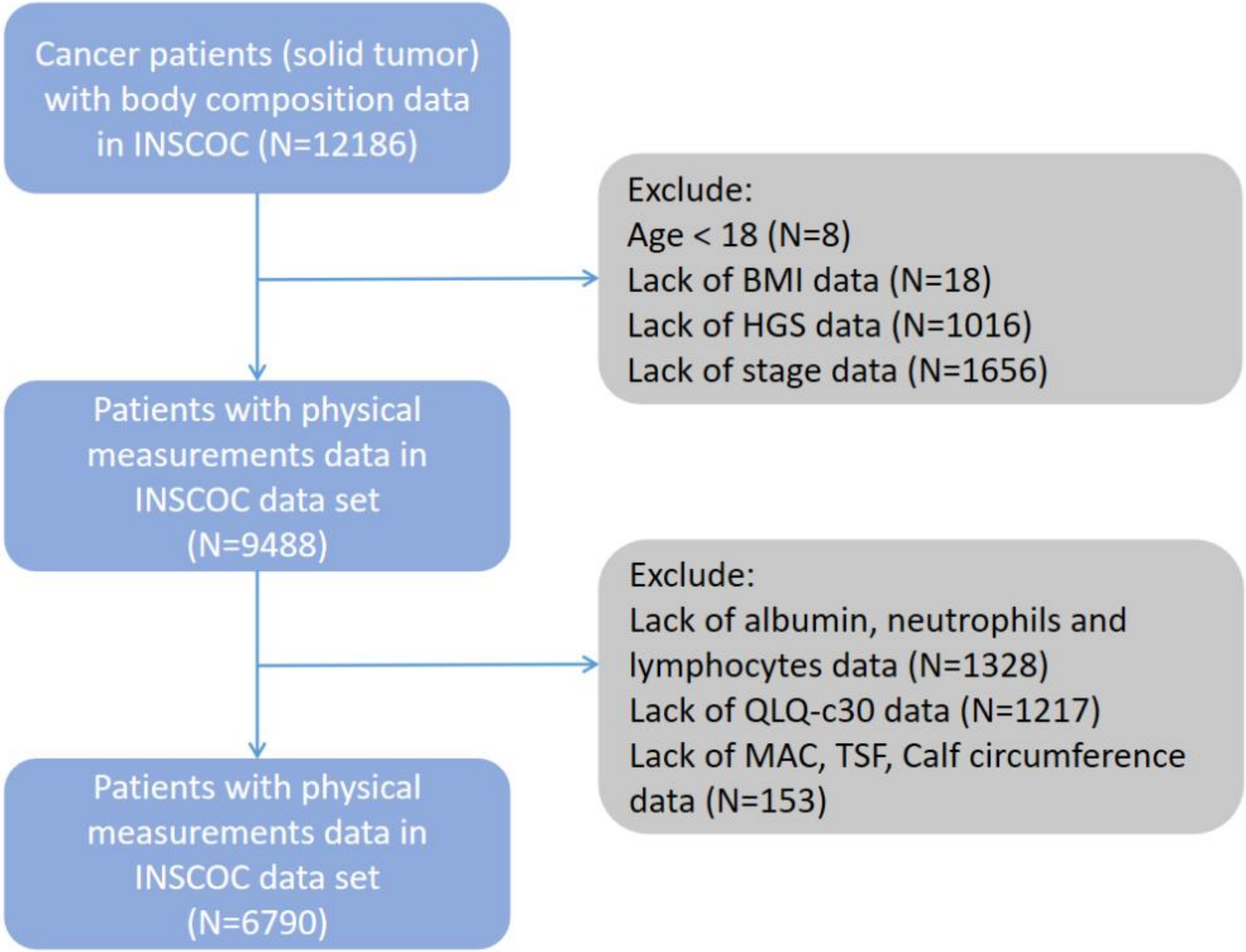

© 2024 Liu C et al. *JAMA Network Open.*

eFigure 2. Prevalence Rate of SO in Patients of Different Ages and Cancer Types

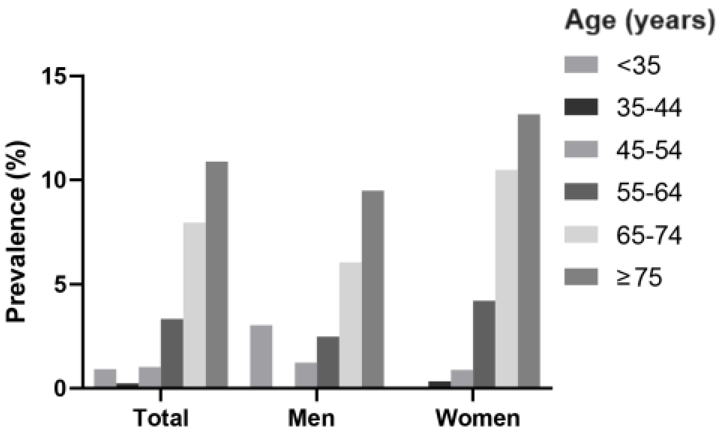

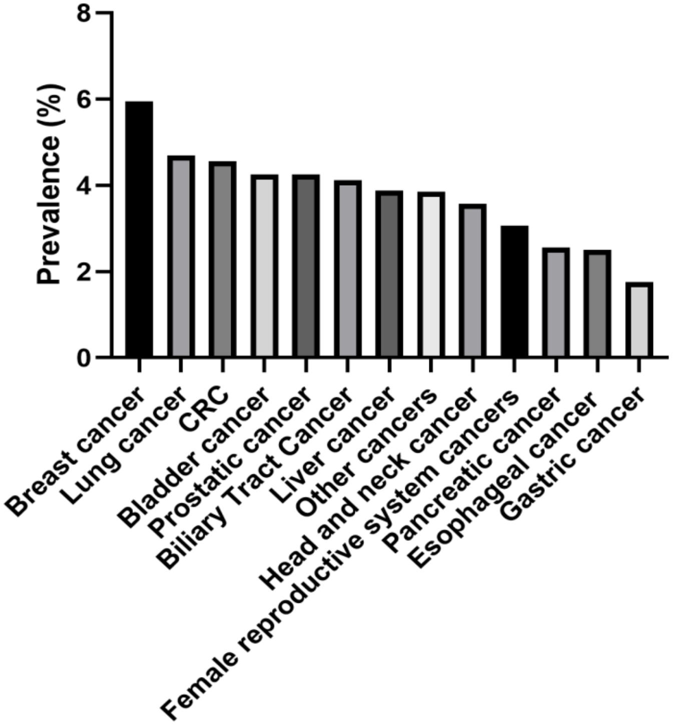

© 2024 Liu C et al. *JAMA Network Open.*

eFigure 3. Subgroup Analysis

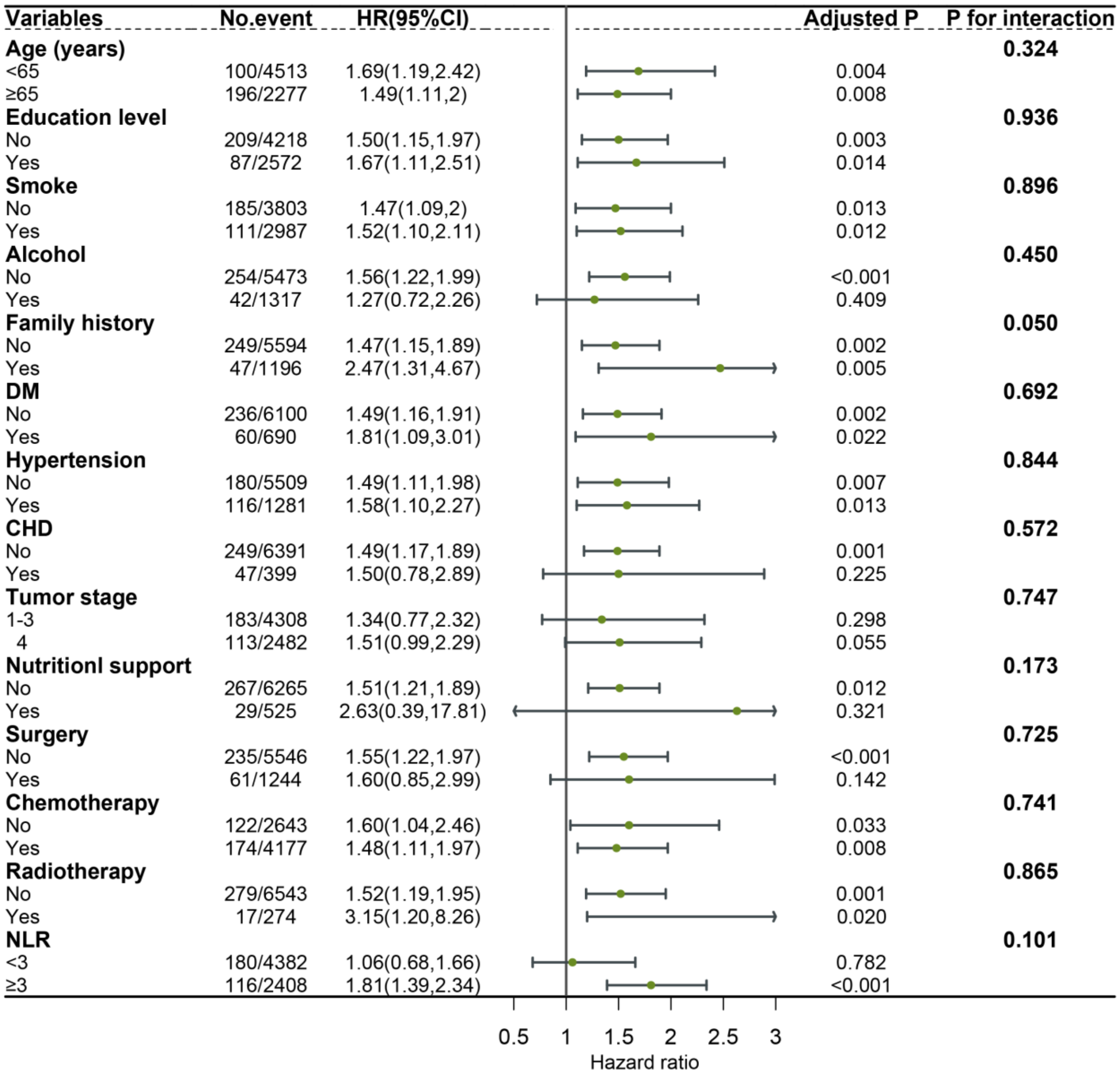

Model was adjusted for age, sex, cancer type, stage, treatment, education level, NLR, family history of cancer, DM, hypertension, CHD, alcohol use, smoke, Scr, Alb, MAC, TSF, calf circumference, weight loss, nutrition support, BMI, HGS, fat mass, ALM/W.

© 2024 Liu C et al. *JAMA Network Open.*

eFigure 4. Kaplan-Meier Curves of OS for Patients Stratified by SO Among Patients With Common Cancer Types

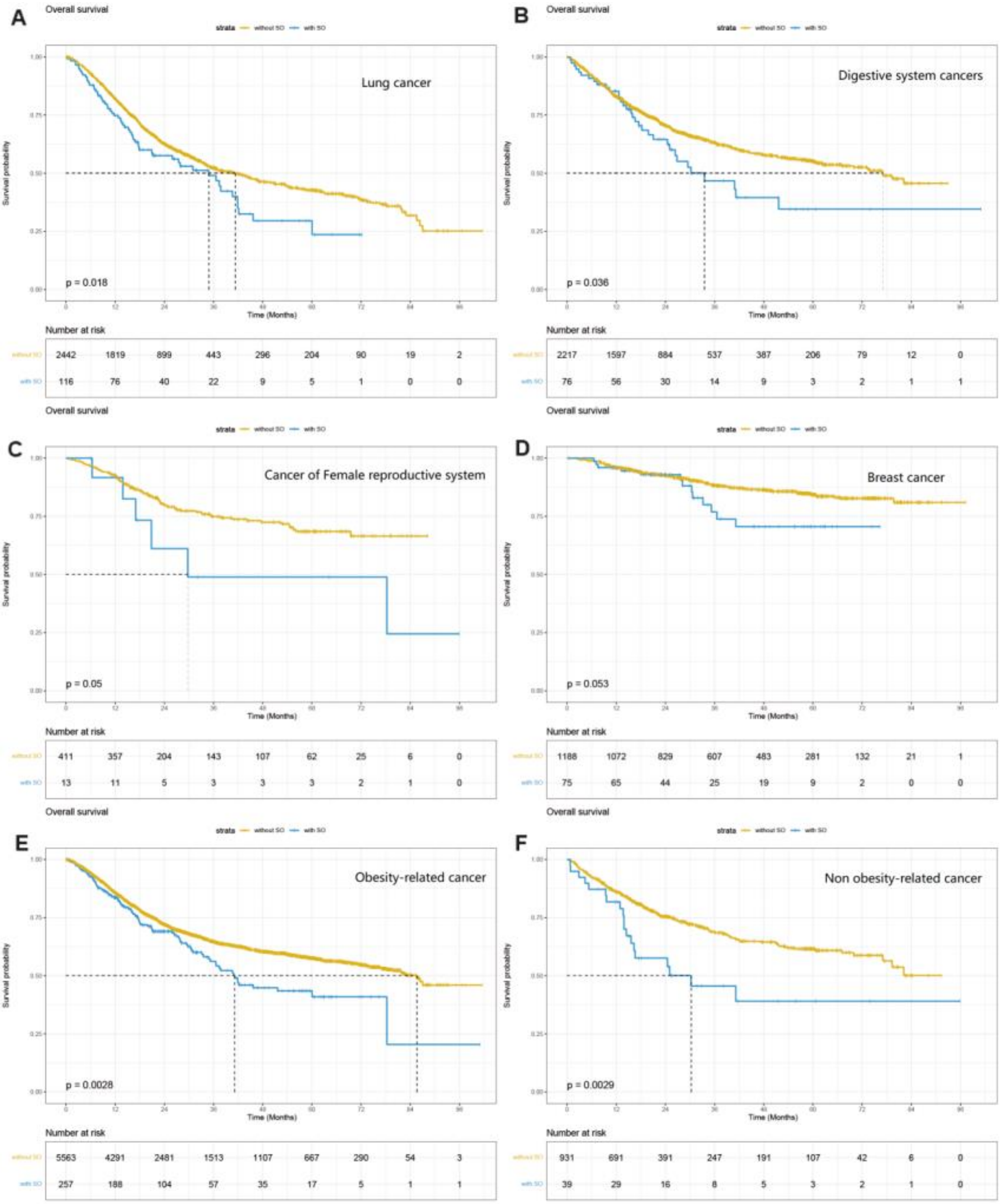

The yellow line represents non-SO patients, while the blue line represents SO patients. eFigure 4A shows the Kaplan-Meier (KM) survival curves for SO in a subgroup of lung cancer patients, B shows the KM curves for SO in a subgroup of gastrointestinal cancer patients, C shows the KM curves for SO in a subgroup of female reproductive system cancer patients, D shows the KM curves for SO in a subgroup of breast cancer patients, E shows the KM curves for SO in a subgroup of obesity-related cancers (esophagus, stomach, colon, rectum, liver, pancreas, lung, breast, corpus uteri, ovaries, prostate, kidney, bladder, brain cancer) patients, and F shows the KM curves for SO in a subgroup of non-obesity-related cancers (other solid tumors not related to obesity) patients.

© 2024 Liu C et al. *JAMA Network Open.*

eFigure 5. Kaplan-Meier Curves of SO and OS in Newly Diagnosed and Treated Patients With Cancer

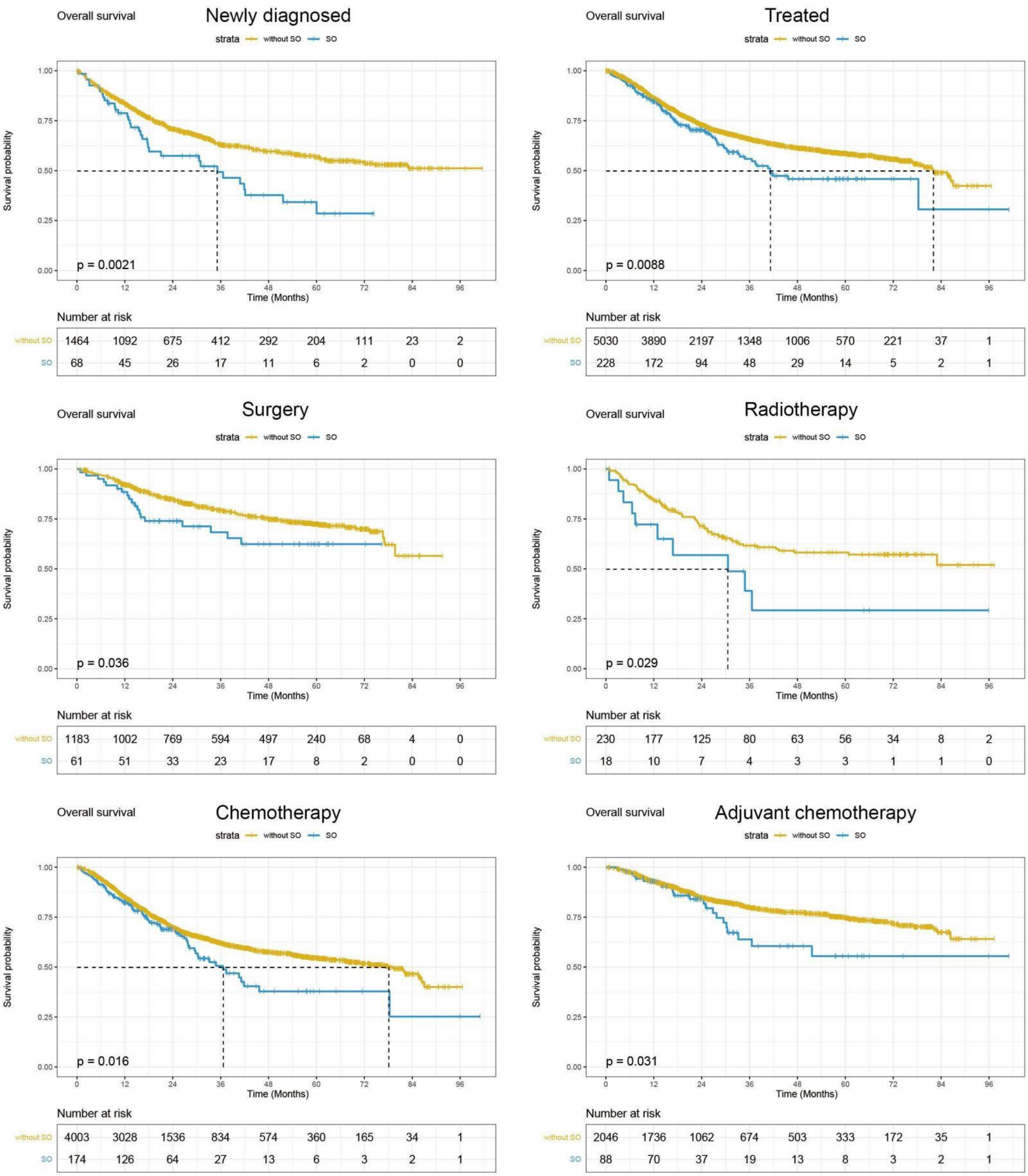

eFigure 6. Kaplan-Meier Curves of OS for Patients Stratified by SO After IPTW

© 2024 Liu C et al. *JAMA Network Open.*

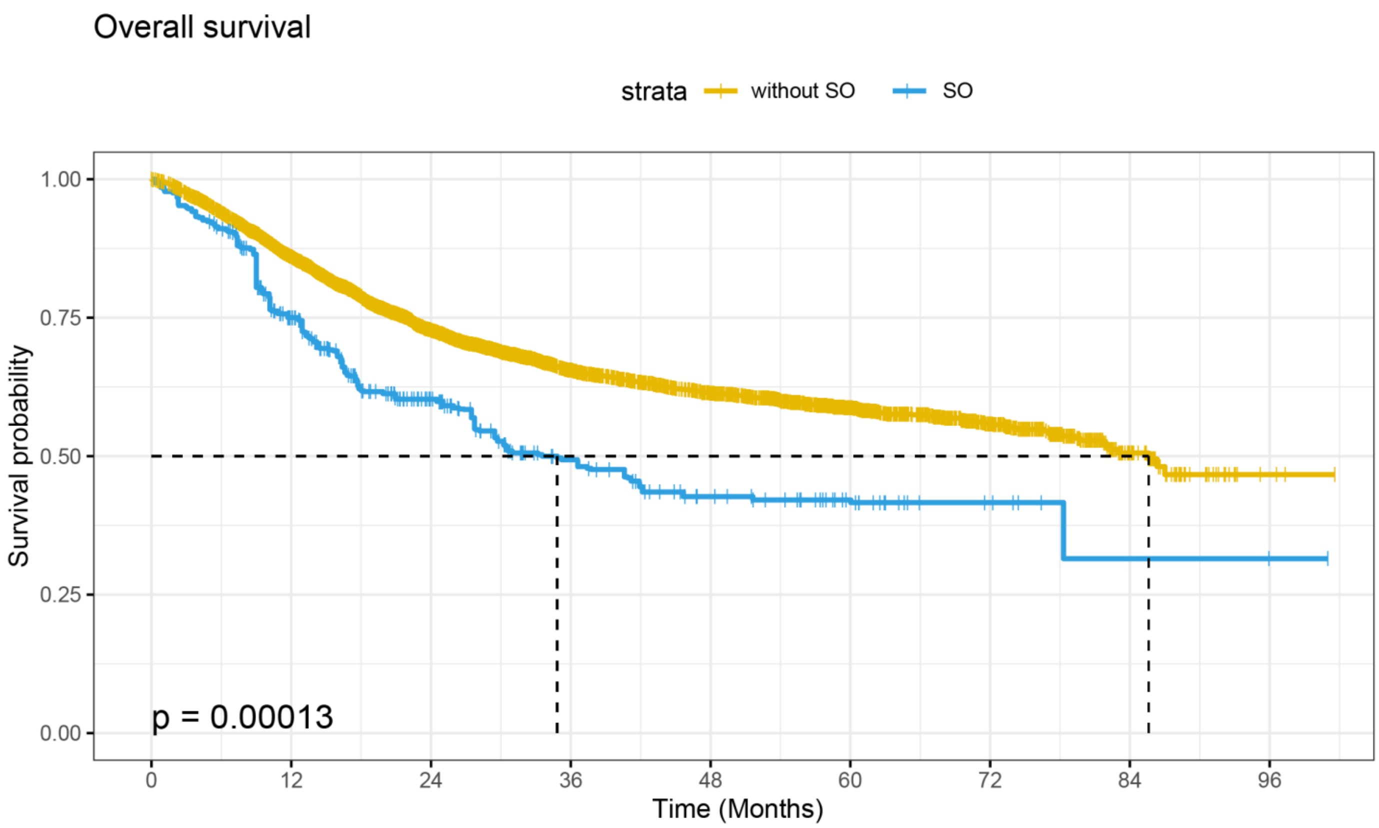

Number at risk

	0	12	24	36	48	60	72	84	96
1	6868	5296	3081	1901	1415	865	346	63	3
2	5193	3577	1757	978	683	280	63	12	2

eFigure 7. Screening, Diagnosis, and Staging Procedure for SO via Cohort-Specific Cutoff

© 2024 Liu C et al. *JAMA Network Open.*

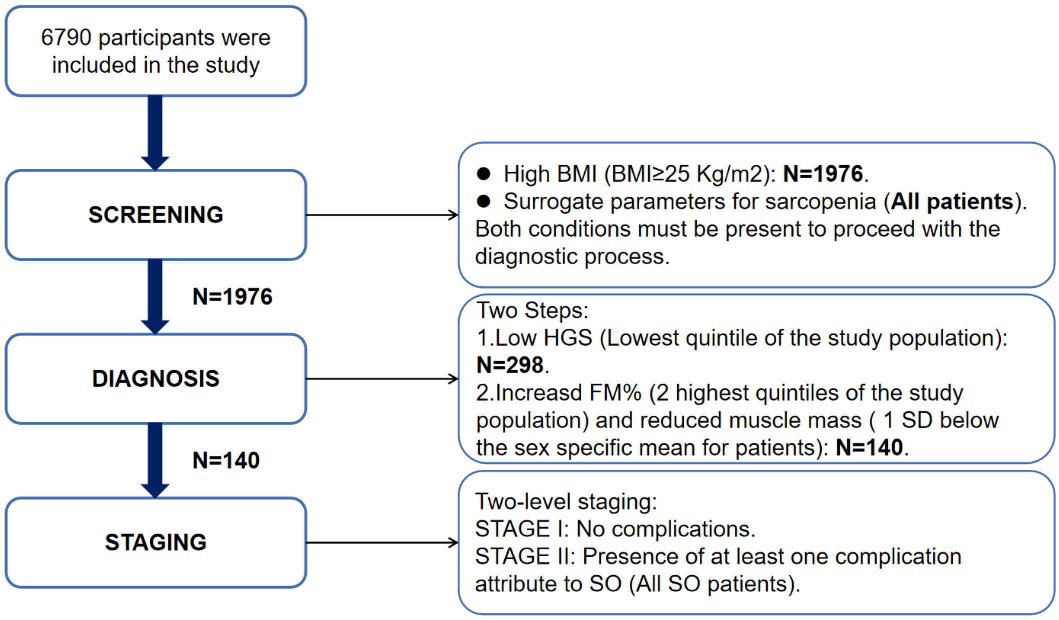

© 2024 Liu C et al. *JAMA Network Open.*

eFigure 8. Prevalence Rate of SO in Patients of Different Ages and Cancer Types (Cohort-Specific Cutoff)

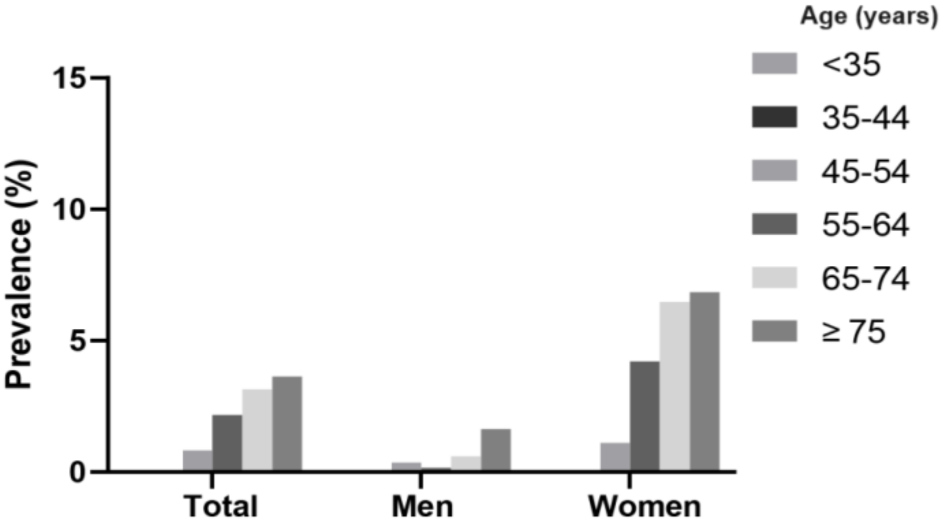

© 2024 Liu C et al. *JAMA Network Open.*

eTable 1. Baseline Characteristics Stratified by SO

	Level	Without SO	With SO	p
N		6494	296	
Age (mean (SD))		59.28 (10.72)	67.61 (8.68)	<0.001
Sex (%)	Men	3172(48.8)	129(43.6)	0.09
	Women	3322 (51.2)	167 (56.4)	
KPS (median [IQR])		90.00 [90.00, 90.00]	90.00 [90.00, 90.00]	0.31
Education level (%)	High school and above	2485 (38.3)	87 (29.4)	0.003
Tumor history (%)	Yes	1149 (17.7)	47 (15.9)	0.47
Diabetes (%)	Yes	630 (9.7)	60 (20.3)	<0.001
Hypertension (%)	Yes	1165 (17.9)	116 (39.2)	<0.001
CHD (%)	Yes	352 (5.4)	47 (15.9)	<0.001
Chronic Liver Disease (%)	Yes	114 (1.8)	4 (1.4)	0.77
COPD (%)	Yes	29 (0.4)	4 (1.4)	0.08
Anemia (%)	Yes	159 (2.4)	3 (1.0)	0.17
Smoke	Yes	2876 (44.3)	122 (41.2)	0.30
Alcohol use (%)	Yes	1275 (19.6)	42 (14.2)	0.03
Stage (%)	1	821 (12.6)	41 (13.9)	0.72
	2	1455 (22.4)	66 (22.3)	
	3	1849 (28.5)	76 (25.7)	
	4	2369 (36.5)	113 (38.2)	
Surgery(%)	Yes	1183 (18.2)	61 (20.6)	0.34
Chemotherapy(%)	Yes	4003 (61.6)	174 (58.8)	0.35
Radiotherapy(%)	Yes	230 (3.5)	18 (6.1)	0.02
NLR (median [IQR])		2.33 [1.55, 3.76]	2.56 [1.69, 3.89]	0.11
Scr (median [IQR])		61.80 [53.20, 72.00]	64.10 [55.27, 74.50]	0.005
Alb (median [IQR])		39.10 [35.60, 42.20]	39.70 [36.10, 42.02]	0.18
MAC (median [IQR])		26.50 [24.50, 28.50]	29.00 [27.00, 31.00]	<0.001
TSF (median [IQR])		17.00 [12.00, 22.00]	22.00 [16.00, 26.00]	<0.001
Calf circumference (median [IQR])		34.00 [31.40, 36.00]	36.00 [34.00, 38.00]	<0.001
Weight loss(%)	Yes	2586 (39.8)	114 (38.5)	0.70
ICU(%)	Yes	1068 (16.4)	58 (19.6)	0.18
Nutrition support(%)	Yes	496 (7.6)	29 (9.8)	0.21
BMI (mean (SD))		22.99 (3.41)	28.36 (2.62)	<0.001
FM (median [IQR])		26.00 [19.70, 32.98]	37.25 [33.45, 42.42]	<0.001
HGS (median [IQR])		23.60 [18.20, 31.00]	16.15 [12.67, 23.10]	<0.001
ALM/W (median [IQR])		29.13 [25.90, 33.18]	24.37 [23.81, 30.31]	<0.001

© 2024 Liu C et al. *JAMA Network Open.*

eTable 2. ***P* value for Bonferroni Correction**

	Non-obese VS Obese Without Sarcopenia	Non-obese VS SO	Obese Without Sarcopenia VS SO
Age (mean (SD))	<0.001	0.008	<0.001
Sex (%)	<0.001	<0.001	0.36
KPS (median [IQR])	<0.001	0.96	0.001
Education level (%)	0.001	0.01	<0.001
Tumor history (%)	0.002	0.74	0.10
Diabetes (%)	<0.001	<0.001	<0.001
Hypertension (%)	<0.001	<0.001	<0.001
CHD (%)	0.003	<0.001	<0.001
Chronic Liver Disease (%)	0.83	0.80	0.72
COPD (%)	0.999	0.09	0.12
Anemia (%)	0.004	0.10	0.71
Smoke	<0.001	0.003	1
Alcohol use (%)	<0.001	0.008	0.41
Stage (%)	<0.001	0.35	0.25
Surgery(%)	0.26	0.27	0.62
Chemotherapy(%)	0.39	0.42	0.24
Radiotherapy(%)	0.65	0.09	0.06
NLR (median [IQR])	<0.001	0.32	0.001
Scr (median [IQR])	0.08	0.003	0.04
Alb (median [IQR])	<0.001	0.004	0.003
MAC (median [IQR])	<0.001	<0.001	0.01
TSF (median [IQR])	<0.001	<0.001	0.75
Calf circumference (median [IQR])	<0.001	<0.001	<0.001
Weight loss(%)	<0.001	0.34	0.26
ICU(%)	0.19	0.13	0.43
Nutrition support(%)	<0.001	0.60	<0.001
BMI (mean (SD))	<0.001	<0.001	<0.001
FM (median [IQR])	<0.001	<0.001	<0.001
HGS (median [IQR])	<0.001	<0.001	<0.001
ALM/W (median [IQR])	<0.001	<0.001	<0.001

© 2024 Liu C et al. *JAMA Network Open.*

eTable 3. Univariate and Multivariate Logistic Regression for Analyzing Associated Factors for SO

	Univariate logistic		Multivariate logistic#	
	OR (95%CI)	P	OR (95%CI)	P
KPS(≥90)	1(0.76,1.31)	0.98	1.22(0.78,1.89)	0.39
Sex	1.24(0.98,1.56)	0.08	2.81(1.63,4.86)	<0.001
Age (≥60y)	5.36(3.87,7.40)	<0.001	4.62(3.01,7.11)	<0.001
Education Level	0.67(0.52,0.87)	0.002	0.46(0.31,0.67)	<0.001
Family History of cancer	0.88(0.64,1.21)	0.42	0.83(0.52,1.33)	0.44
DM	2.37(1.76,3.18)	<0.001	1.33(0.81,2.19)	0.26
Hypertension	2.95(2.32,3.75)	<0.001	1.45(0.98,2.13)	0.06
CHD	3.29(2.37,4.58)	<0.001	1.50(0.87,2.59)	0.14
Chronic Liver Disease	0.81(0.30,2.22)	0.69	1.32(0.42,4.10)	0.63
Chronic Kidney Disease	2.69(1.06,6.87)	0.04	1.91(0.59,6.19)	0.28
COPD	3.25(1.13,9.32)	0.03	2.01(0.53,7.71)	0.31
Anemia	0.43(0.14,1.36)	0.15	0.75(0.22,2.57)	0.64
Smoke	1.33(1.04,1.69)	0.02	1.60(1.07,2.37)	0.02
Alcohol Use	0.68(0.49,0.94)	0.02	0.74(0.41,1.32)	0.31
Advanced Stage	1.29(1.02,1.64)	0.03	1.47(1.02,2.10)	0.04
Surgery	1.17(0.87,1.59)	0.31	1.13(0.50,2.55)	0.78
Chemotherapy	0.92(0.70,1.20)	0.52	1.03(0.69,1.53)	0.91
Radiotherapy	2.05(1.26,3.35)	0.004	4.98(2.46,10.07)	<0.001
Hypoalbuminemia	0.62(0.33,1.17)	0.14	0.38(0.10,1.41)	0.15
Nutrition Support	1.20(0.80,1.81)	0.37	0.59(0.14,2.53)	0.48
Weight Loss	0.95(0.75,1.20)	0.65	1.55(1.08,2.22)	0.02
NLR (≥3)	1.35(1.06,1.73)	0.02	1.13(1.01,1.58)	0.04
MAC*	1.11(1.07,1.17)	<0.001	0.97(0.91,1.05)	0.45
TSF*	1.04(1.02,1.06)	<0.001	1.02(0.97,1.07)	0.37
Calf circumference*	1.07(1.03,1.10)	<0.001	1.02(0.97,1.07)	0.47
TC*	1.08(0.98,1.19)	0.14	1.01(0.88,1.16)	0.88
TG*	1.03(0.97,1.10)	0.27	0.99(0.88,1.12)	0.89

*These variables were all logarithmically transformed

Model was adjusted for age, sex, cancer type, stage, treatment, education level, NLR, family history of cancer, DM, hypertension, CHD, alcohol use, smoke, Scr, Alb, MAC, TSF, calf circumference, weight loss, nutrition support, BMI, HGS, fat mass, ALM/W.

© 2024 Liu C et al. *JAMA Network Open.*

eTable 4. The Association Between SO and Outcomes Stratified by Cancer Types and Sex

	All			Men			Women		
	E/T	HR (95%CI)	P	E/T	HR (95%CI)	P	E/T	HR (95%CI)	P
Lung cancer	120/2558	1.49 (1.09,2.03)	0.01	68/1590	1.44 (0.95,2.18)	0.08	48/968	1.35 (0.83,2.19)	0.23
Esophageal cancer	7/279	0.90 (0.24,3.41)	0.87	7/260	1 (0.25,4.06)	0.99	0/19	NA	
Gastric cancer	10/568	1.66 (0.21,12.97)	0.63	6/389	NA		4/179	1.78 (0.18,18.02)	0.62
Colorectal cancer	45/987	2.19 (1.13,4.25)	0.02	26/589	1.32 (0.39,4.54)	0.66	19/398	3.81 (1.55,9.34)	0.003
Liver cancer	8/206	2.69 (0.81,8.95)	0.11	6/156	3.34 (0.77,14.44)	0.11	2/50	8.81 (0.10,762.03)	0.34
Pancreatic cancer	3/156	15.60 (11.59,34.67)	<0.001	2/87	NA		1/69	NA	
Bile and extrahepatic bile duct tumors	4/97	NA		2/55	NA		2/42	NA	
Breast cancer	75/1263	1.52 (0.76,3.02)	0.24	2/4	NA		73/1259	1.52 (0.76,3.02)	0.24
Bladder cancer	2/47	NA		1/36	NA		1/11	NA	
Prostatic cancer	3/47	NA		3/47	NA		NA/NA	NA	
Head and neck cancer	1/28	NA		0/21	NA		1/7	NA	
Cancer of female reproductive system	13/424	2.99 (0.84,10.68)	0.09	NA	NA		13/424	2.99 (0.84,10.68)	0.09
Other cancers	5/130	NA			NA			NA	
Digestive system cancer	76/2293	1.53 (1.07,2.18)	0.02	48/1536	1.47 (0.93,2.34)	0.10	28/757	1.65 (0.95,2.88)	0.08
Non digestive system cancer	220/4497	1.58 (1.22,2.04)	<0.001	81/1765	1.63 (1.11,2.40)	0.01	139/2732	1.43 (1.01,2.03)	0.046

Model was adjusted for age, sex, cancer type, stage, treatment, education level, NLR, family history of cancer, DM, hypertension, CHD, alcohol use, smoke, Scr, Alb, MAC, TSF, calf circumference, weight loss, nutrition support, BMI, HGS, fat mass, ALM/W.

E/T: SO events/ Total number of patients with this tumor type

© 2024 Liu C et al. *JAMA Network Open.*

eTable 5. Sensitivity Analyses

Excluding patients with follow-up less than 1 year	All (N=6218)		Men (N=2957)		Women (N=3261)	
	HR (95%CI)	P	HR (95%CI)	P	HR (95%CI)	P
Model 0	1.45 (1.18,1.79)	<0.001	1.34 (0.99,1.80)	0.06	1.67 (1.25,2.23)	<0.001
Model 1	1.58 (1.27,1.97)	<0.001	1.35 (0.99,1.83)	0.054	1.58 (1.18,2.13)	0.002
Model 2	1.47 (1.12,1.91)	0.005	1.40 (0.95,2.05)	0.09	1.59 (1.13,2.23)	0.007
Excluding patients with BMI>35kg/m^2	All (N=6772)		Men (N=3298)		Women (N=3474)	
Model 0	1.45 (1.20,1.75)	<0.001	1.38 (1.07,1.79)	0.01	1.60 (1.22,2.10)	0.001
Model 1	1.43 (1.18,1.73)	<0.001	1.38 (1.06,1.80)	0.02	1.48 (1.12,1.95)	0.006
Model 2	1.57 (1.25,1.97)	<0.001	1.54 (1.11,2.14)	0.009	1.53 (1.12,2.10)	0.008
Additionally adjusted for squared age term	All (N=6790)		Men (N=3301)		Women (N=3489)	
Model 3	1.44 (1.20,1.74)	<0.001	1.38 (1.06,1.79)	0.02	1.50 (1.14,1.96)	0.004
Model 4	1.74 (1.43,2.12)	<0.001	1.71 (1.31,2.25)	<0.001	1.72 (1.30,2.28)	0.001
Additionally adjusted for time-varying covariates	All (N=6790)		Men (N=3301)		Women (N=3489)	
Model 5	1.39 (1.17,1.70)	<0.001	1.27 (1.03,1.72)	0.02	1.55 (1.19,2.01)	<0.001

Model 0 was the crude model.

Model 1 was adjusted for age, sex, cancer type, stage, treatment.

Model 2 was adjusted for age, sex, cancer type, stage, treatment, education level, NLR, family history of cancer, DM, hypertension, CHD, alcohol use, smoke, Scr, Alb, MAC, TSF, calf circumference, weight loss, nutrition support, BMI, HGS, fat mass, ALM/W.

Model 3 was adjusted for age, age square, sex, cancer type, stage, treatment.

Model 4 was adjusted for age, age square, sex, cancer type, stage, treatment, education level, NLR, family history of cancer, DM, hypertension, CHD, alcohol use, smoke, Scr, Alb, MAC, TSF, calf circumference, weight loss, nutrition support, BMI, HGS, fat mass, ALM/W.

Model 5 was adjusted for age (time-varying), sex (time-varying), cancer type, stage (time-varying), treatment (time-varying), education level, NLR (time-varying), family history of cancer, DM (time-varying), hypertension (time-varying), CHD (time-varying), alcohol use (time-varying), smoke (time-varying), Scr (time-varying), Alb (time-varying), MAC (time-varying), TSF (time-varying), calf circumference (time-varying), weight loss (time-varying), nutrition support (time-varying), BMI (time-varying), HGS (time-varying), fat mass (time-varying), ALM/W (time-varying).

© 2024 Liu C et al. *JAMA Network Open.*

eTable 6. Baseline Characteristics After IPTW

	Level	Non-obese	Obese		p
			Obese Without Sarcopenia	SO	
N		5046	1822	5193	
Age (mean (SD))		60.32 (11.19)	60.02 (10.04)	60.41 (10.55)	0.41
Sex (%)	Men	2595 (51.4)	729 (40.0)	2599 (50.0)	0.11
	Women	2451 (48.6)	1093 (60.0)	2594 (50.0)	
KPS (median [IQR])		90.00[80.00,90.00]	90.00[90.00,100.00]	90.00 [90.00, 90.00]	<0.001
Education level (%)	High school and above	1881 (37.3)	754 (41.4)	1840 (35.4)	0.40
Family history of tumor (%)	Yes	833 (16.5)	365 (20.0)	928 (17.9)	0.49
Diabetes (%)	Yes	454 (9.0)	251 (13.8)	726 (14.0)	0.04
Hypertension (%)	Yes	1129 (22.4)	388 (21.3)	1123 (21.6)	0.53
CHD (%)	Yes	270 (5.4)	151 (8.3)	358 (6.9)	0.15
Chronic Liver Disease (%)	Yes	86 (1.7)	32 (1.8)	87 (1.7)	0.96
COPD (%)	Yes	23 (0.5)	8 (0.4)	73 (1.4)	0.04
Anemia (%)	Yes	138 (2.7)	27 (1.5)	99 (1.9)	0.53
Smoke	Yes	2329 (46.2)	673 (36.9)	2639 (50.8)	0.05
Alcohol use (%)	Yes	1030 (20.4)	289 (15.8)	992 (19.1)	0.44
Stage (%)	1	597 (11.8)	308 (16.9)	719 (13.8)	0.38
	2	1085 (21.5)	449 (24.6)	1139 (21.9)	
	3	1467 (29.1)	469 (25.7)	1235 (23.8)	
	4	1896 (37.6)	597 (32.8)	2100 (40.4)	
Surgery(%)	Yes	941 (18.6)	354 (19.4)	1012 (19.5)	0.85
Chemotherapy(%)	Yes	3051 (60.5)	1130 (62.0)	3256 (62.7)	0.68
Radiotherapy(%)	Yes	181 (3.6)	61 (3.3)	429 (8.3)	0.01
NLR (median [IQR])		2.43 [1.58, 3.90]	2.17 [1.50, 3.38]	2.31 [1.61, 3.76]	<0.001
Scr (median [IQR])		61.70 [53.30, 71.80]	62.40 [53.80, 73.00]	64.60 [54.44, 75.98]	0.045

© 2024 Liu C et al. *JAMA Network Open.*

Alb (median [IQR])		38.70 [35.10, 41.90]	40.40 [37.10, 43.30]	39.79 [35.50, 42.36]	<0.001
MAC (median [IQR])		26.00 [24.00, 27.50]	29.40 [28.00, 31.00]	28.00 [26.00, 30.19]	<0.001
TSF (median [IQR])		16.00 [12.00, 20.00]	22.00 [17.00, 26.00]	18.08 [14.85, 25.00]	<0.001
Calf circumference (median [IQR])		33.00 [31.00, 35.00]	36.70 [34.80, 39.00]	35.00 [32.50, 37.00]	<0.001
Weight loss(%)	Yes	2071 (41.0)	649 (35.6)	2176 (41.9)	0.41
ICU(%)	Yes	851 (16.9)	324 (17.8)	1012 (19.5)	0.002
Nutrition support(%)	Yes	448 (8.9)	96 (5.4)	486 (9.4)	0.29
BMI (mean (SD))		21.52 (2.32)	27.39 (2.16)	28.30 (2.30)	<0.001
FM (median [IQR])		23.70 [17.90, 30.10]	34.10 [27.80, 38.39]	35.94 [31.70, 42.09]	<0.001
HGS (median [IQR])		23.00 [17.52, 30.20]	24.90 [19.90, 33.14]	16.59 [12.60, 23.55]	<0.001
ALM/W (median [IQR])		31.67 [26.51, 33.80]	25.77 [24.83, 30.90]	26.42 [24.09, 30.60]	<0.001

Abbreviation: ALM/W, appendicular lean mass adjusted for body weight, BMI, body mass index, FM fat mass, HGS, hand grip strength, KPS, Karnofsky Performance Score, MAC, maximum upper arm circumference,,NLR, neutrophil-to-lymphocyte ratio, Scr, serum creatinine, SO, sarcopenic obesity, TSF, triceps skinfold thickness.

© 2024 Liu C et al. *JAMA Network Open.*

eTable 7. Associations Between SO and OS After IPTW

	All		Men		Women	
	HR (95%CI)	P	HR (95%CI)	P	HR (95%CI)	P
Model 0	1.66(1.20,2.29)	0.002	1.27(1.01,1.85)	0.04	1.96(1.11,3.47)	0.02
Model 1	1.88(1.36,2.60)	<0.001	1.60(1.12,2.28)	0.009	1.90(1.22,2.97)	0.005
Model 2	2(1.42,2.82)	<0.001	1.77(1.12,2.81)	0.02	1.72(1.26,2.34)	0.001

Model 0 was the crude model.

Model 1 was adjusted for age, sex, cancer type, stage, treatment.

Model 2 was adjusted for age, sex, cancer type, stage, treatment, education level, NLR, family history of cancer, DM, hypertension, CHD, alcohol use, smoke, Scr, Alb, MAC, TSF, calf circumference, weight loss, nutrition support, BMI, HGS, fat mass, ALM/W.

© 2024 Liu C et al. *JAMA Network Open.*

eTable 8. QLQc30 for Patients With SO and Patients Without SO

	Without SO	SO	P
	6494	296	
QLQ-c30 domains			
Functioning scales			
Physical functioning	85.86 (18.96)	81.34 (20.58)	<0.001
Role functioning	85.10 (23.27)	80.58 (23.84)	0.008
Emotional functioning	89.81 (16.07)	87.07 (18.29)	0.02
Cognitive functioning	89.43 (16.04)	86.17 (18.72)	0.006
Social functioning	79.41 (26.19)	78.35 (27.16)	0.58
Cancer-related symptom scales			
Fatigue	17.90 (21.12)	21.42 (22.58)	0.02
Nausea and vomiting	4.16 (12.19)	2.84 (9.71)	0.14
Pain	11.29 (19.32)	13.83 (18.95)	0.07
Dyspnea	10.92 (19.94)	14.78 (23.49)	0.009
Insomnia	15.87 (24.99)	18.73 (24.91)	0.12
Anorexia	9.97 (20.62)	8.42 (16.02)	0.30
Constipation	6.01 (15.88)	4.82 (15.54)	0.30
Diarrhea	3.65 (13.09)	2.41 (9.88)	0.19
Financial difficulties	29.31 (30.87)	28.17 (31.41)	0.62
Global health status	70.28 (21.58)	66.54 (21.57)	0.02

© 2024 Liu C et al. *JAMA Network Open.*

eTable 9. Association Between SO and Risk of ICU Admission

	Before IPTW		After IPTW	
	OR (95%CI)	P	OR (95%CI)	P
Model 0	1.37 (1.03, 1.83)	0.03	1.43 (1.09,1.94)	0.002
Model 1	1.29 (0.94, 1.75)	0.11	1.39 (1,1.82)	0.054
Model 2	2.39 (1.06, 5.29)	0.04	2.55 (1.18,5.41)	0.001

Model 0 was the crude model.

Model 1 was adjusted for age, sex, cancer type, stage, treatment.

Model 2 was adjusted for age, sex, cancer type, stage, treatment, education level, NLR, family history of cancer, DM, hypertension, CHD, alcohol use, smoke, Scr, Alb, MAC, TSF, calf circumference, weight loss, nutrition support, BMI, HGS, fat mass, ALM/W.

© 2024 Liu C et al. *JAMA Network Open.*

eTable 10. Associations Between SO (Cohort-Specific Cutoff) and its Components and OS

	All		Men		Women	
	HR (95%CI)	P	HR (95%CI)	P	HR (95%CI)	P
	SO		SO		SO	
Model 0	1.48 (1.15,1.91)	0.002	1.35 (0.64,2.84)	0.43	1.48 (1.15,1.91)	0.002
Model 1	1.86 (1.40,2.47)	<0.001	1.22 (0.46,3.27)	0.69	1.86 (1.40,2.47)	<0.001
Model 2	1.98 (1.49,2.64)	<0.001	1.33 (0.49,3.62)	0.57	1.98 (1.45,2.70)	<0.001
	BMI		BMI		BMI	
Model 0	0.83 (0.75,0.91)	<0.001	0.94 (0.83,1.07)	0.37	0.81 (0.70,0.94)	0.004
Model 1	0.86 (0.78,0.96)	0.007	0.85 (0.73,0.99)	0.04	0.89 (0.76,1.04)	0.13
Model 2	1.10 (0.96,1.25)	0.16	1.01 (0.85,1.21)	0.89	1.22 (1.01,1.48)	0.04
	FM		FM		FM	
Model 0	0.98 (0.89,1.06)	0.57	0.99 (0.88,1.11)	0.87	0.96 (0.84,1.10)	0.54
Model 1	0.87 (0.79,0.96)	0.005	0.89 (0.78,1.01)	0.07	0.89 (0.76,1.03)	0.11
Model 2	1.01 (0.91,1.13)	0.79	0.99 (0.86,1.14)	0.88	1.07 (0.91,1.26)	0.42
	HGS		HGS		HGS	
Model 0	1.65 (1.50,1.82)	<0.001	1.4 (1.23,1.60)	<0.001	2 (1.73,2.31)	<0.001
Model 1	1.44 (1.29,1.61)	<0.001	1.27 (1.09,1.48)	0.002	1.66 (1.41,1.94)	<0.001
Model 2	1.22 (1.09,1.37)	0.001	1.11 (0.95,1.30)	0.20	1.33 (1.13,1.58)	0.001
	ALM/W		ALM/W		ALM/W	
Model 0	0.83 (0.74,0.94)	0.003	1.01 (0.76,1.35)	0.93	1.11 (0.96,1.28)	0.15
Model 1	0.97 (0.84,1.13)	0.73	0.85 (0.61,1.18)	0.33	0.95 (0.80,1.13)	0.57
Model 2	1.17 (1,1.37)	0.047	1.12 (0.78,1.59)	0.54	1.15 (0.95,1.38)	0.15

Model 0 was the crude model.

Model 1 was adjusted for age, sex, cancer type, stage, treatment.

Model 2 was adjusted for age, sex, cancer type, stage, treatment, education level, NLR, family history of cancer, DM, hypertension, CHD, alcohol use, smoke, Scr, Alb, MAC, TSF, calf circumference, weight loss, nutrition support, BMI, HGS, fat mass, ALM/W.

© 2024 Liu C et al. *JAMA Network Open.*
