# Supplementary material for: Sarcopenic Obesity and Outcomes for Patients With Cancer
Source: JAMA Netw Open. 2024 Jun 14;7(6):e2417115. doi: 10.1001/jamanetworkopen.2024.17115 (PMC11179127; doi:10.1001/jamanetworkopen.2024.17115)
Supplement: Supplement 2. — Data Sharing Statement [file jamanetwopen-e2417115-s002.pdf]

Data Sharing Statement

Liu. Sarcopenic Obesity and Outcomes for Patients With Cancer. *JAMA Netw Open*. Published June 14, 2024. doi:10.1001/jamanetworkopen.2024.17115

Data
Data available: No
